# Plasma Chemical Looping: Unlocking High-Efficiency CO_2_ Conversion to Clean CO at Mild Temperatures

**DOI:** 10.1021/jacsau.4c00153

**Published:** 2024-05-08

**Authors:** Yanhui Long, Xingzi Wang, Hai Zhang, Kaiyi Wang, Wee-Liat Ong, Annemie Bogaerts, Kongzhai Li, Chunqiang Lu, Xiaodong Li, Jianhua Yan, Xin Tu, Hao Zhang

**Affiliations:** † State Key Laboratory of Clean Energy Utilization, Zhejiang University, Hangzhou 310027, China; ‡ College of Energy Engineering, ZJU-UIUC, Zhejiang University, Hangzhou 310027, China; § School of Mechanical Engineering, Shanghai Jiao Tong University, Shanghai 200240, China; ∥ Research Group PLASMANT, Department of Chemistry, University of Antwerp, Universiteitsplein 1, Antwerp 2610, Belgium; ⊥ State Key Laboratory of Complex Nonferrous Metal Resources Clean Utilization, Kunming University of Science and Technology, Kunming 650093, China; # Department of Electrical Engineering and Electronics, University of Liverpool, Liverpool L69 3GJ, U.K.; ∇ Ningbo Innovation Center, Zhejiang University, Ningbo 315100, China

**Keywords:** gliding arc, chemical looping, CO_2_ conversion, oxygen carrier

## Abstract

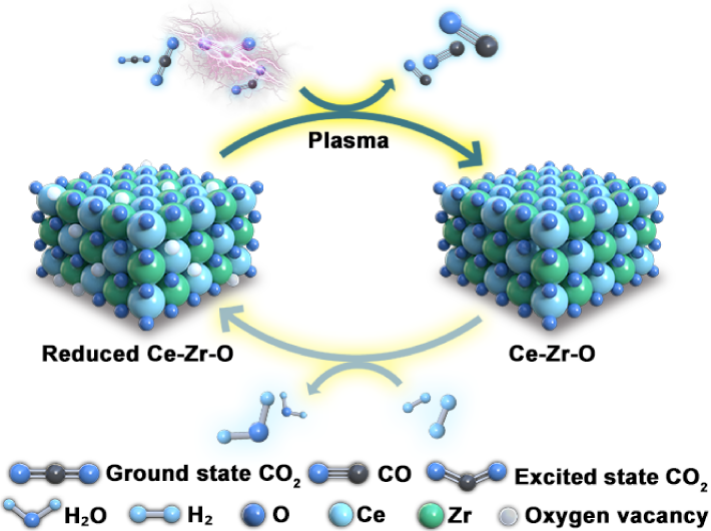

We propose a plasma chemical looping CO_2_ splitting (PCLCS) approach that enables highly efficient CO_2_ conversion into O_2_-free CO at mild temperatures. PCLCS achieves an impressive 84% CO_2_ conversion and a 1.3 mmol g^–1^ CO yield, with no O_2_ detected. Crucially, this strategy significantly lowers the temperature required for conventional chemical looping processes from 650 to 1000 °C to only 320 °C, demonstrating a robust synergy between plasma and the Ce_0.7_Zr_0.3_O_2_ oxygen carrier (OC). Systematic experiments and density functional theory (DFT) calculations unveil the pivotal role of plasma in activating and partially decomposing CO_2_, yielding a mixture of CO, O_2_/O, and electronically/vibrationally excited CO_2_*. Notably, these excited CO_2_* species then efficiently decompose over the oxygen vacancies of the OCs, with a substantially reduced activation barrier (0.86 eV) compared to ground-state CO_2_ (1.63 eV), contributing to the synergy. This work offers a promising and energy-efficient pathway for producing O_2_-free CO from inert CO_2_ through the tailored interplay of plasma and OCs.

## Introduction

The escalating reliance on fossil fuels has led to a significant surge in anthropogenic carbon dioxide (CO_2_) emissions, surpassing a record level of 410 ppm.^1^ Confronted with this pressing environmental challenge, there is a compelling imperative to address and curtail CO_2_ emissions by harnessing it as a valuable C1-feedstock through various chemical processes.^2,3^ One such attractive process is the dissociation of CO_2_ into carbon monoxide (CO), a pivotal industrial feedstock essential for the synthesis of various chemicals and fuels, including alcohols, liquid hydrocarbons, and organic acids.^4,5^ This avenue of carbon utilization represents an appealing means to close the carbon loop, potentially opening up innovative avenues for the chemical industry.^4^

Nevertheless, owing to the linear structure and chemical inertia of CO_2_, this reaction faces a formidable thermodynamic barrier (CO_2_ → CO + 1/2 O_2_, Δ*H*_298 K_ = 280 kJ mol^–1^) in breaking the C=O bond (803 kJ mol^–1^). Despite many advances and successful proof-of-concepts being reported, the activation of CO_2_ for effective conversion remains a great challenge.^3,6−9^ For thermal and photothermal processes, CO_2_ splitting becomes favorable only at extremely elevated temperatures, with the reaction equilibrium strongly favoring the formation of reactants. Even at temperatures as high as 2000 K, the conversion of CO_2_ reaches only 1.5%.^10−12^ Electrochemical methods grapple with issues of low energetic efficiency (or large overpotential), and sluggish electron transfer kinetics.^13^ Similarly, photochemical processes face limitations in terms of photon efficiency.^3^ Nonthermal plasmas (NTPs) have recently emerged as a promising avenue for CO_2_ conversion under mild condition, attributed to the abundance of reactive species (electrons, radicals, and excited species).^6,8,12,14^ Moreover, plasma processes ensure rapid startup, high reaction rates, compactness, ease of installation, and flexibility. These attributes enable the direct utilization of electricity generated from intermittent renewable sources, offering a flexible solution for peak shaving and grid stabilization.^12^ Nonetheless, challenges in conversion and energy efficiency constrain their potential application.^12^

Furthermore, the typically inevitable presence of O_2_ in the gas products not only hinders CO_2_ conversion, particularly due to recombination reactions at elevated gas temperatures, but also introduces the risk of catalyst deactivation.^11−13,15^ For instance, electrocatalytic conditions with rapid electron collections typically have an O_2_ tolerance of less than 2% v/v.^16^ Certainly, the necessity for an expensive gas separation/purification process to eliminate O_2_ impurities represents another significant challenge.^17^

To address the above challenges, we propose a novel plasma chemical looping CO_2_ splitting (PCLCS) approach to efficiently convert CO_2_ into O_2_-free CO. This approach, integrating a custom-built rotating gliding arc (RGA) plasma with a reduced oxygen carrier (OC), harnesses the strong activation capabilities of plasma on inert CO_2_ molecules^6^ and the distinctive oxygen-carrying capacity of OCs for effective CO_2_ reduction.^18^ The RGA provides a stable “warm” plasma with a high energy density and allows for instant on/off for the initial activation and partial composition of CO_2_. Significantly, compared to conventional NTPs such as dielectric barrier discharge (DBD), the energy distribution within RGA stimulates the most efficient CO_2_ decomposition route via vibrational excitation. Zirconium-doped ceria (Ce_*x*_Zr_1–*x*_O_2_, *x* = 0.1–0.5) forming a Ce–Zr–O solid solution was developed as the OC. Compared to other typical OCs (e.g., Fe-based, Mn-based), ceria is particularly attractive owing to its rapid redox kinetics and robust structural and crystallographic stability.^18^ In addition, the addition of Zr^4+^ into CeO_2_ could considerably enhance the surface/bulk oxygen mobility and reactivity by introducing crystallographic defects.^19^

The efficacy of the PCLCS system was appraised over Ce_*x*_Zr_1–*x*_O_2_ with varying Zr contents, and a comprehensive examination of the physicochemical characteristics of the OCs was undertaken. Notably, achieving a CO_2_ conversion of up to 84% and a CO yield of 1.3 mmol g^–1^, devoid of detectable O_2_, at mild temperature of only 320 °C in the OC region highlights a robust synergy between plasma and the OC. The intricate interplay between the plasma and the OC was meticulously elucidated through a synergistic combination of extensive experimental analyses and molecular scale density functional theory (DFT) calculations. The decisive contribution of excited CO_2_*, generated by the plasma, and its interactions with the oxygen vacancies of the OC were convincingly demonstrated as a pivotal factor driving the observed synergy.

## Results and Discussion

### Performance of PCLCS

**Figure 1:**
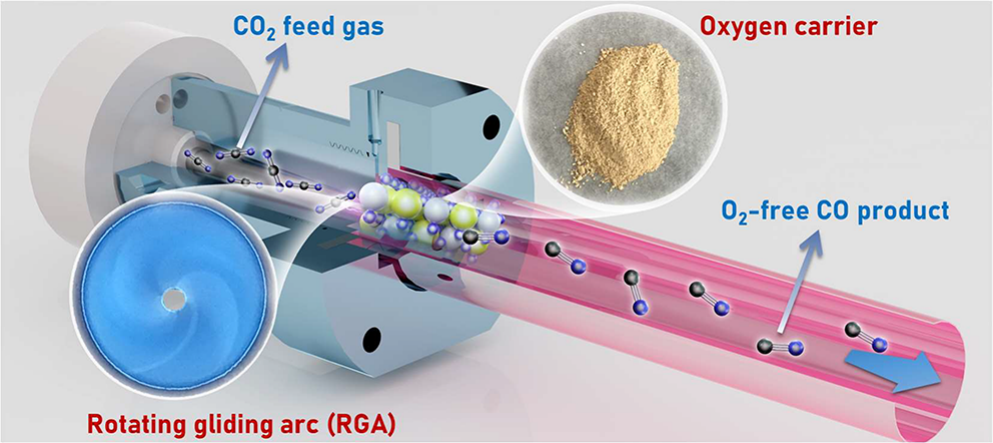
Schematic of the Plasma Chemical Looping CO_2_ Splitting (PCLCS) setup.

The PCLCS setup comprising a custom-built RGA reactor and a quartz cover housing the OCs is illustrated in Figure 1. Further information on the setup configuration and the experimental system is presented in Figures S1 and S2 of Section S1. PCLCS experiments were conducted at a weight hourly space velocity (WHSV) of 300,000 cm^3^ g^–1^ h^–1^ without external heating.

**Figure 2:**
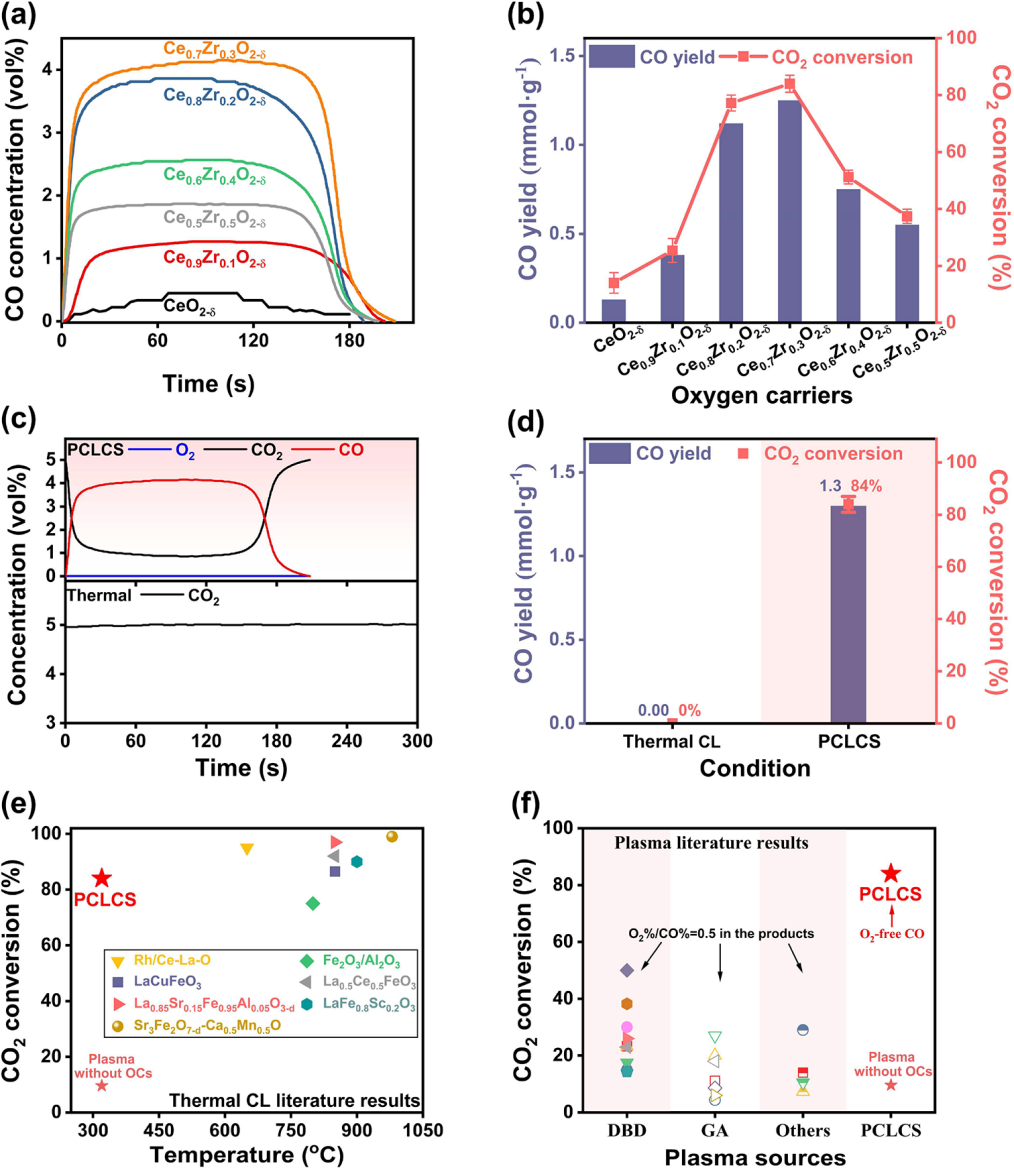
Performance of PCLCS. (a) Time-resolved concentrations of CO and (b) CO_2_ conversion and CO yield over Ce_1–*x*_Zr_*x*_O_2−δ_ (x = 0, 0.1, 0.2, 0.3, 0.4, and 0.5). Comparison of PCLCS (over reduced Ce_0.7_Zr_0.3_O_2−δ_) and thermal chemical looping (CL) at 320 °C: (c) Time-resolved product composition, (d) CO yield and CO_2_ conversion, and (e) CO_2_ conversion with respect to the reaction temperature among PCLCS and state-of-the-art thermal CL CO_2_ splitting works.^20−26^ (f) CO_2_ conversion among PCLCS and state-of-the-art NTP for CO_2_ splitting.^6,7,27−45^

The time-resolved CO concentrations in the products, as well as the CO_2_ conversion and CO yield of PCLCS using different Ce_1–*x*_Zr_*x*_O_2−δ_ OCs, are plotted in Figure 2. More detailed concentration profiles are provided in Figures S3 and S4. For Zr-doped OCs, CO is immediately produced upon plasma activation, reaching its maximum concentration within only 5 to 20 s, maintaining for approximately 110–140 s, and followed by a rapid decline to zero due to the oxidation of the OCs. CeO_2−δ_ exhibits a significantly lower reaction rate, as indicated by the low CO concentration and its slow initial increase rate. Interestingly, no O_2_ was detected in the gas products.

Incorporating Zr into CeO_2_ significantly enhances the reaction performance, with effects varying depending on the doping ratio. Figure 2a,b, shows a consistent improvement in both the CO concentration (and yield) and the CO_2_ conversion as the Zr content increases from 0% to 30%. Ce_0.7_Zr_0.3_O_2−δ_, in particular, achieves the highest CO_2_ conversion (up to ∼84%), a substantial increase compared to the limited CO_2_ conversion of CeO_2−δ_ (12%). Consequently, Ce_0.7_Zr_0.3_O_2−δ_ attains a maximum CO yield of 1.3 mmol g^–1^, surpassing the yield of CeO_2−δ_ by a factor of around seven. However, a further increase in the Zr content to 50% leads to a significant decline in reaction performance. These trends align well with the OC characterization results in Table 1, namely that the oxygen vacancy abundance of the OCs reaches a maximum at a Zr content of 30%, followed by a remarkable drop as the Zr content further increases to 50%. Based on these results, Ce_0.7_Zr_0.3_O_2−δ_ was chosen as the OC for subsequent experiments.

**Table 1:** XPS-Derived Characteristics of Ce_1–*x*_Zr_*x*_O_2(-δ)_ OCs

		oxygen species percentage (%)				Ce ion percentage (%)		
oxygen carriers		O I	O II	O III	O_ads_/O_latt_ ratio	Ce^3+^	Ce^4+^	Ce^3+^/Ce^4+^ ratio
fresh	CeO_2_	58	38	4	0.72	11.97	88.02	0.14
	Ce_0.9_Zr_0.1_O_2_	48	41	11	1.08	14.62	85.38	0.17
	Ce_0.8_Zr_0.2_O_2_	40	28	32	1.50	15.85	84.15	0.19
	Ce_0.7_Zr_0.3_O_2_	27	61	12	2.70	21.14	78.86	0.27
	Ce_0.6_Zr_0.4_O_2_	41	43	16	1.44	13.52	86.48	0.16
	Ce_0.5_Zr_0.5_O_2_	55	35	10	0.82	11.17	88.83	0.13
reduced	CeO_2−δ_	28	62	10	2.57	13.97	86.03	0.16
	Ce_0.7_Zr_0.3_O_2−δ_	10	86	4	9.00	34.67	65.33	0.53
oxidized	CeO_2_	57	37	6	0.75	11.02	88.98	0.12
	Ce_0.7_Zr_0.3_O_2_	24	67	9	2.03	20.80	79.20	0.26

Importantly, these results were achieved at a mild reaction temperature of only 320 °C on the OCs, as measured by the thermocouple. To elucidate the specific role of plasma in CO_2_ conversion in PCLCS, thermal chemical looping (CL) CO_2_ splitting experiments over reduced Ce_0.7_Zr_0.3_O_2−δ_ were comparatively performed in a tube furnace at the same temperature of 320 °C. Figure 2c–f presents a comparison of the time-resolved product composition, CO yield, and CO_2_ conversion between thermal CL experiments and PCLCS. Additionally, we compare the performance of PCLCS with typical CO_2_ splitting results reported in literature for NTP and thermal CL processes.

As shown in Figure 2c,d, the thermal CL method fails to reduce CO_2_ at such a mild temperature of 320 °C. In contrast, PCLCS, aided by plasma without additional heating, achieves a remarkable CO_2_ conversion of up to 84% and a CO yield of 1.3 mmol g^–1^. The CO_2_ temperature program oxidation (CO_2_-TPO) results presented in Figure S5 reveal that thermal CL CO_2_ splitting is initiated only at above 400 °C. A further comparison with state-of-the-art literature results (Figure 2e) shows that PCLCS dramatically reduces the required reaction temperature compared to thermal CL from 650 to 1000 to 320 °C, with comparable or only slightly lower CO_2_ conversion. Moreover, PCLCS achieves significantly higher CO_2_ conversion (∼84%) compared to typical NTP sources in the literature (only 4%–50%), as shown in Figure 2f. The energy efficiency of PCLCS (6.4% to 18.2%, refer to Table S1) is comparable or superior to that of DBD plasmas (typically <10%), although slightly lower than that of gliding arc (GA) or other warm plasmas (typically <35%).^12^ Nonetheless, further enhancement is anticipated through the utilization of more OCs or optimization of the reaction conditions, as suggested by the results in Table S1. Note that no O_2_ was detected in the products of our PCLCS system, while in NTP processes, an O_2_ concentration of half that of CO is typically reported.^12,33^

The above results suggest that PCLCS provides a strong synergy between plasma and OCs in facilitating the kinetics of CO_2_ splitting, achieving high CO_2_ conversion (∼84%), and an O_2_-free CO product (1.3 mmol g^–1^), at a mild temperature of only 320 °C. It should be noted that the oxidized OC requires reduction at a relatively high temperature in a tube furnace (800 °C under H_2_ in this work). However, there are avenues for lowering the reaction temperature. For instance, modifying Ce-based OCs with trace amounts of metals such as Rh and Ni, or utilizing Mo-based OCs, as demonstrated in previous literature,^20,46,47^ have shown promise in reducing the required reaction temperature.

The stability of the PCLCS system and the OCs was further assessed through 10 redox cycle tests. Results in Figure S5 show that the CO yield and CO_2_ conversion remained consistently high, around 1.3 mmol g^–1^ and 82–84%, respectively, throughout the cycles. The minimal degradation, only approximately 0.038% per cycle, demonstrated excellent redox stability. Moreover, the CO purity in the products remained almost 100%, with no detectable presence of O_2_. The physicochemical characteristics of the Ce_0.7_Zr_0.3_O_2_ OCs before (fresh) and after (cycled) the redox cycle are presented in Figures S7 and S8. The X-ray diffraction (XRD) patterns in Figure S7a confirm that both the fresh and cycled samples exhibit crystal structures consistent with the standard fluorite type cubic phase of CeO_2_ (JCPDS card [43–1002]). The H_2_-temperature programmed reduction (H_2_-TPR) profile of the cycled Ce_0.7_Zr_0.3_O_2_ in Figure S7b closely resembles that of the fresh sample, indicating complete restoration of the crystallite state after the redox cycle. Scanning electron microscopy (SEM) and transmission electronmicroscopy (TEM) images in Figures S7c,d and S8 depict that the Zr element region almost coincides with the Ce element regions in the fresh sample, suggesting a uniform dispersion of Zr cations in the CeO_2_ lattice (Figure S7d). After cycling, all elements exhibit a similar distribution with no detectable agglomeration of Zr or other elements. In addition, the SEM images show only a negligible increase in the particle size for the cycled sample. All these observations collectively indicate that the microstructure of Ce_0.7_Zr_0.3_O_2_ OCs remains stable during the redox cycles, substantially contributing to the high stability of PCLCS for O_2_-free CO production from CO_2_.

### Physicochemical Characteristics of the OCs: Effect of Zr Doping

To unravel the underlying mechanisms of PCLCS and comprehend the distinct performance exhibited by various OCs, we conducted a comprehensive examination of the physicochemical characteristics of Ce_1–*x*_Zr_*x*_O_2_, highlighting the impact of Zr doping. Moreover, DFT calculations were performed to gain insight into the effects at an atomic level.

The XRD patterns of the freshly synthesized Ce_1–*x*_Zr_*x*_O_2_ (*x* = 0, 0.1, 0.2, 0.3, 0.4, 0.5) OCs are presented in Figure S9, revealing that the crystal structures of all samples match well the face-centered cubic (fcc) fluorite structure of CeO_2_, with no detectable impurity phases, except in the case of Ce_0.5_Zr_0.5_O_2_ (Figure S9a). The enlarged portion of single CeO_2_ in Figure S9b shows that the diffraction peaks of CeO_2_ (111) in Zr-containing samples shift slightly toward higher diffraction angles, accompanied by peak broadening. To scrutinize the structural changes in the fluorite structure after Zr doping, Rietveld refinement calculations were performed on the XRD patterns of CeO_2_ and Ce_0.7_Zr_0.3_O_2_ samples, as an example, as presented in Figure S9c,d and summarized in Table S2. The table summarizes that the R_wp_ value for both samples is below 10%, and the χ^2^ value is around 3.0, indicating the high reliability of the refined results due to the good match between the theoretical model and the test data. It can be inferred that Zr doping in the CeO_2_ lattice is achieved by replacing some of Ce^4+^. In this case, the cell parameters (a, b, and c) of Ce_0.7_Zr_0.3_O_2_ are slightly smaller than those of CeO_2_ due to the smaller Zr^4+^ radius (0.08 nm) compared to Ce^4+^ (0.092 nm). The results suggest that the Zr ions are incorporated into the CeO_2_ lattice to form a Ce–Zr–O solid solution.

H_2_-TPR experiments were conducted to assess the oxygen mobility of Ce_1–*x*_Zr_*x*_O_2_. As depicted in Figure S10, CeO_2_ exhibits two distinct reduction peaks, with the first spanning 490 to 600 °C, and the second consistently rising after 700 °C.^19,48,49^ The low-temperature peak can be attributed to the consumption of surface oxygen species on CeO_2_, while the high temperature peak involves the consumption of bulk lattice oxygen and the reduction of Ce^4+^ to Ce^3+^. Increasing Zr content (*x* = 0.1, 0.2, 0.3, 0.4) shifts the second peak to lower temperatures and the first peak to higher temperatures, forming a larger peak. This shift indicates simultaneous surface and bulk reduction. As surface oxygen is consumed, bulk oxygen rapidly migrates to replenish the depleted surface oxygen, reflecting the reducibility and mobility of lattice oxygen.^50,51^ These results indicate that Zr introduction into CeO_2_ enhances lattice oxygen reduction at lower temperatures, indicating significantly improved lattice oxygen mobility. Among these six samples, Ce_0.7_Zr_0.3_O_2_ exhibits the largest peak area and highest H_2_ consumption, suggesting that 30% Zr doping is optimal for achieving a desired balance between active CeO_2_ and inert ZrO_2_.

**Figure 3:**
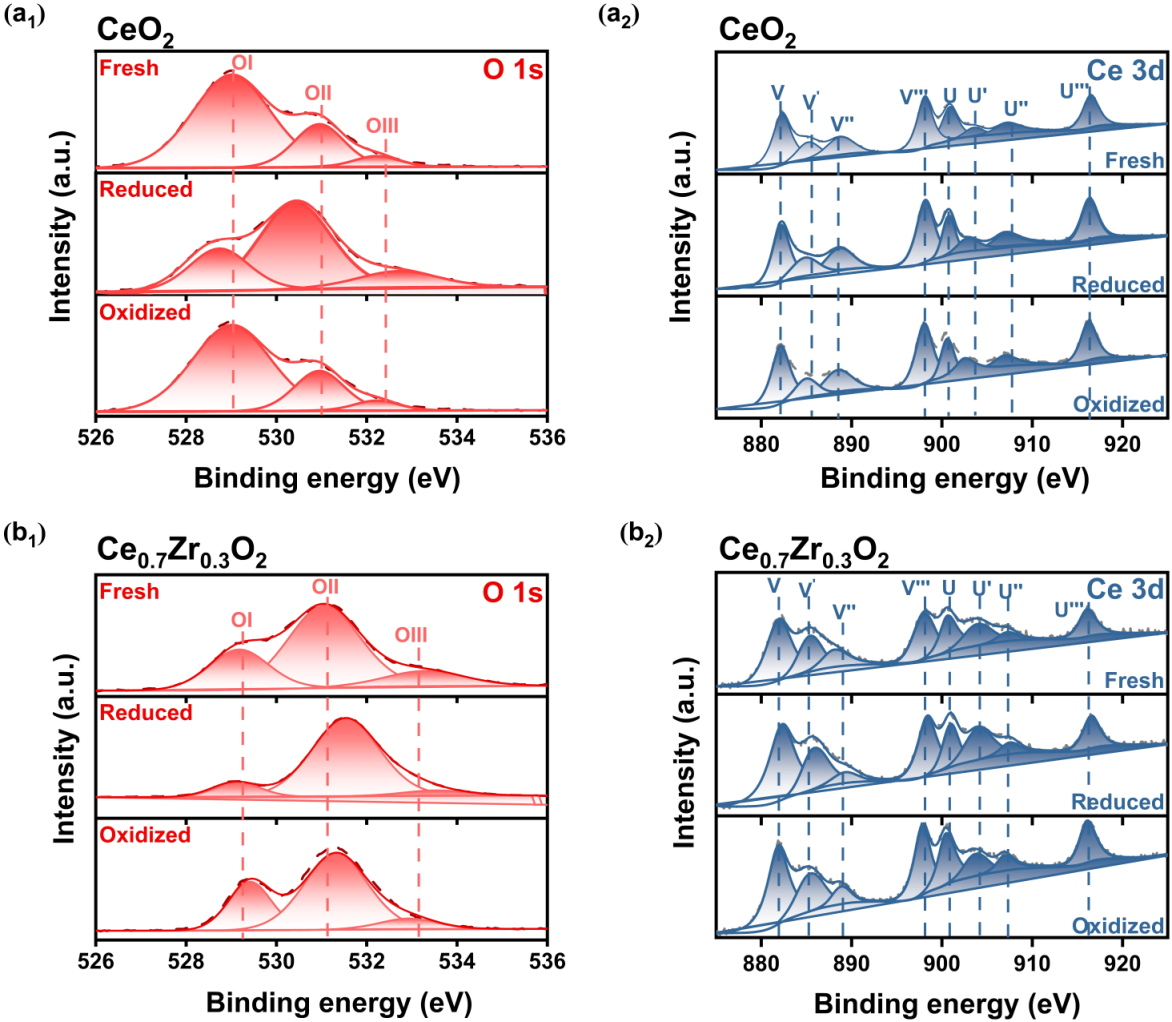
XPS results. XPS spectra for O 1s and Ce 3d over (a) CeO_2_ and (b) Ce_0.7_Zr_0.3_O_2_ at various stages (fresh, reduced, and oxidized).

To elucidate the surface elemental composition and chemical status of the OCs in various states, X-ray photoelectron spectroscopy (XPS) characterization was conducted on freshly prepared Ce_1–*x*_Zr_*x*_O_2_ (labeled as “fresh”) with different Zr contents (*x* = 0, 0.1, 0.2, 0.3, 0.4, 0.5). The corresponding spectra are presented in Figure S11. Additionally, CeO_2_ and Ce_0.7_Zr_0.3_O_2_, used in our PCLCS experiments, was characterized at different stages: 1) fresh; 2) reduced before use in PCLCS (labeled as “reduced”); and 3) oxidized after use in PCLCS (labeled as “oxidized”), with the spectra illustrated in Figure 3. In the O 1s spectrum (Figures S11a and 3a), the predominant peak at 529.5 eV (labeled as O I) corresponds to lattice oxygen (O_latt_), while the minor peaks at 531.8 eV (labeled as O II) and 533.2 eV (labeled as O III) likely represent low coordination surface-absorbed oxygen species (O_ads_, e.g., hydroxyl and carbonate species).^52−54^ In the Ce 3d spectrum (Figures S11b and 3b), V (V, V′, V″, and V‴) peaks correspond to Ce 3d_5/2_, and U (U, U′, U″, and U‴) peaks represent Ce 3d_3/2_. The V–U, V″–U″, and V‴–U‴ doublets indicate Ce (IV) final states (Ce^4+^ ions), while V′ and U′ peaks suggest Ce^3+^ ions.^54,55^ The atomic molar ratios of the surface species across different samples, calculated based on the fitted peaks, are tabulated in Table 1. Considering that Brunauer−Emmett−Teller (BET) characterization shows no notable difference in the specific surface area for different OCs (e.g., CeO_2_: 36.6 m^2^ g^–1^ vs Ce_0.7_Zr_0.3_O_2_: 37.3 m^2^ g^–1^), the concentration of oxygen vacancies in OCs can be considered as proportional to the O_ads_/O_latt_ ratio.^54,56^ Zr to CeO_2_ enhances the O_ads_/O_latt_ ratio, as summarized in Table 1. Nevertheless, after reaching a maximum for Ce_0.7_Zr_0.3_O_2_ (2.70), the ratio of the O_ads_/O_latt_ then drops with further rising Zr content from x = 0.3 to 0.5. This indicates that adding an appropriate amount of Zr enhances the formation of oxygen vacancies, likely due to the chemical interactions between ZrO_2_ and CeO_2_. Consistently, Table 1 summarizes that the introduction of Zr^4+^ increases the Ce^3+^/Ce^4+^ ratio in Ce_1–*x*_Zr_*x*_O_2_, inducing the formation of oxygen vacancies. Again, Ce_0.7_Zr_0.3_O_2_ exhibited the highest Ce^3+^ concentration, implying the most abundant oxygen vacancies. For higher Zr content (*x* ≥ 0.4), the reduced oxygen vacancy concentration in Ce_0.6_Zr_0.4_O_2_ and Ce_0.5_Zr_0.5_O_2_ is likely attributed to the ZrO_2_ agglomeration on the material and the limited solid solution content (as observed in the XRD patterns in Figure S9).^57^

The H_2_-TPR and XPS results reveal that Ce_0.7_Zr_0.3_O_2_ possesses the most abundant oxygen vacancies, aligning well with the experimental findings (see Figure 2a,b) where Ce_0.7_Zr_0.3_O_2_ demonstrated the highest CO yield and CO_2_ conversion. This underscores the pivotal role of oxygen vacancy abundance in PCLCS. Additionally, the characterization results for CeO_2_ and Ce_0.7_Zr_0.3_O_2_ at different stages in Table 1 indicate that reducing the OCs significantly improves the oxygen vacancies, with the reduced Ce_0.7_Zr_0.3_O_2−δ_ showing remarkably higher abundance. This contributes significantly to the efficient conversion of CO_2_ in PCLCS. Above all, the concentration of oxygen vacancies plays a virtual role in the PCLCS process.

To elucidate the role of Zr in a Ce–Zr–O solid solution at an atomic level, DFT calculations were conducted to determine (i) the bulk and surface oxygen vacancy formation energies (Δ*E*_V_) and (ii) the energy barrier for oxygen vacancy migration in both pure and Zr-doped ceria (CeO_2_ and Ce_0.7_Zr_0.3_O_2_, respectively). The former quantifies the thermodynamic energy required for lattice oxygen removal, and the latter depicts the ease of vacancy migration from a kinetics viewpoint. Both are crucial factors for the oxygen conductivity that is required for macroscopic lattice oxygen removal and redeposition in redox reactions. Moreover, Bader charge of CeO_2_ and Ce_0.7_Zr_0.3_O_2_ that refer to the simulated valence electron and electronic projected density of states (PDOS) of CeO_2−δ_ and Ce_0.7_Zr_0.3_O_2−δ_ were studied to further illustrate the bonding ability between certain surface atoms of the substrate. The computational details are provided in Section S2.

**Figure 4:**
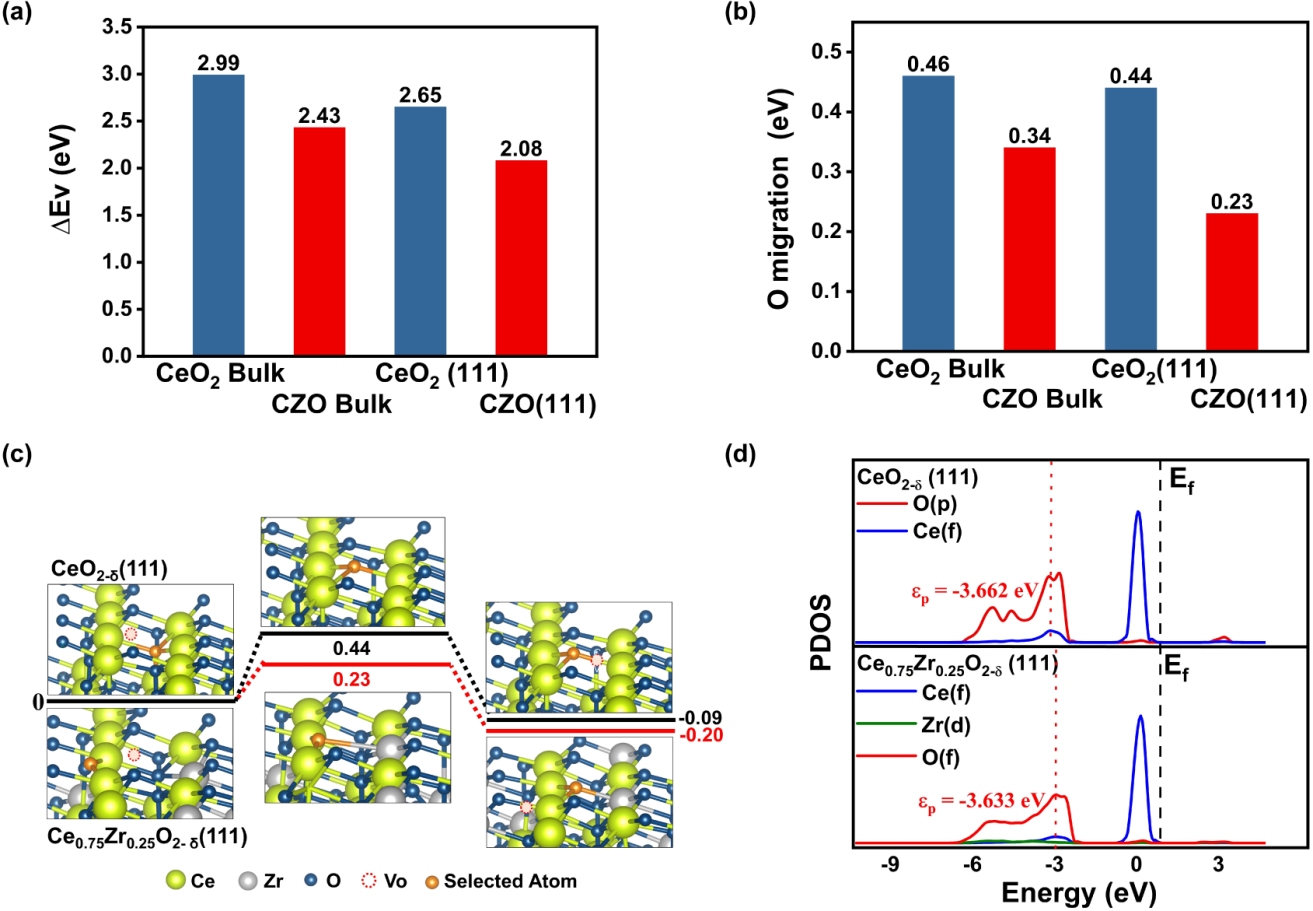
Effect of Zr doping. (a) Computed energies of oxygen vacancy formation (Δ*E*_*V*_) and (b) Energy barriers of oxygen vacancy migration, for bulk and (111) surface of CeO_2_ and Ce_0.7_Zr_0.3_O_2_ (CZO). (c) Energy potential profile along the most favorable oxygen migration pathway on the (111) surfaces of CeO_2_ and Ce_0.7_Zr_0.3_O_2_ (CZO). (d) Electronic projected density of states (PDOS) of CeO_2−δ_ and Ce_0.7_Zr_0.3_O_2−δ_.

In the construction of the Ce_0.7_Zr_0.3_O_2_ unit cell, two Ce atoms in the pristine CeO_2_ unit cell were replaced by Zr atoms, and then, one O atom was removed to form an oxygen vacancy. These model structure details are presented in Section S2.1. The main DFT calculation results are presented in Figure 4.

The computed bulk oxygen vacancy formation energy (Δ*E*_*V*_) for Ce_0.7_Zr_0.3_O_2_ was found to be 2.43 eV (Figure 4a), notably lower than that for pristine CeO_2_ (2.99 eV.^58^ Additionally, the ease of O migration in Ce_0.7_Zr_0.3_O_2_ bulk can be correlated with a lower barrier of 0.34 eV compared to 0.46 eV for pure CeO_2_^59^ (Figure 4b), as computed through transition-state calculations. For vacancy creation and migration on the surface, the (111) surface was selected for both CeO_2_ and Ce_0.7_Zr_0.3_O_2_ since it is reported to be the most stable ceria surface.^20,60^ Indeed, many studies indicate that doping with various elements can substantially modify the surface properties of CeO_2_.^19,20,59,61^ A smaller Δ*E*_V_ of 2.08 eV (Figure 4a) was computed for the Ce_0.7_Zr_0.3_O_2_ (111) surface than for the Ce_0.7_Zr_0.3_O_2_ bulk (2.43 eV) (Figure 4a), indicating a higher surface oxygen vacancy concentration than the initially assumed Ce_0.7_Zr_0.3_O_2_ stoichiometry. The migration pathway of the subsurface oxygen to the vacancy site has a very low barrier of 0.23 eV, which is much lower than that of the CeO_2_ (111) surface (0.44 eV) (Figure 4c). More detailed calculation methods and results about oxygen formation energy and migration barrier calculations of CeO_2_ and Ce_0.75_Zr_0.25_O_2−δ_ (111) surface or bulk are presented in Section S2.1 and Section S2.2.

Overall, the DFT results indicate significantly easier oxygen migration and release in Zr-doped ceria. The doping of Zr can effectively reduce the electron donation by the cations compared to CeO_2_, thereby decreasing the number of valence electrons on oxygen anions (from 1.22 ^e–^ per O for pristine ceria to 1.20 ^e–^ according to the Bader charge analysis results). The reduction in electron density weakens the strength of the metal–oxygen (M-O) ionic bond. This effect of Zr substitution, combined with existing oxygen vacancies, reorganizes the electronic states in Ce_0.7_Zr_0.3_O_2_ compared to those in CeO_2_. Such a reorganization of oxygen electronic states can be well described by the oxygen p-band center (ε_p_), which is related to the hybridization of metal and oxygen orbitals and has been used as an effective descriptor of the activity of lattice oxygen. Figure 4d shows an upward shift of ε_p_ after Zr doping, indicating stronger coupling between the electronic orbitals of oxygen and metal atoms. This enhanced coupling contributes to the higher activity of lattice oxygen in Ce_0.7_Zr_0.3_O_2_, making Zr doping an effective strategy to improve the redox performance of ceria by reducing the energy barriers for both oxygen vacancy formation and migration.

### Synergy Between Plasma and OC

In this section, we investigate the underlying synergy mechanisms through a combination of experiments and theoretical calculations. In the plasma-OC tandem system, the reactant CO_2_ initially traverses the plasma region and subsequently interacts with the OCs. The plasma, generating highly energetic electrons and excited species, activates and partially splits CO_2_, as previously reported.^6,11,62^ This process produces excited CO_2_*, besides ground-state CO_2_, as well as CO, O_2_, and O radicals, which undergo further CO_2_ conversion and O_2_/O removal on the OC, facilitated by the abundance of oxygen vacancies.

To gain insights into the reactions occurring in these two stages (ground stage and excited stage), we measured the intermediate gas products after the plasma (before the OC) utilizing a meticulously designed in situ gas sampling setup positioned between the plasma and the OC. This sampling setup allows for “freezing” the chemical composition of the sampled plasma gas products, thereby minimizing secondary reactions during sampling. Details of the sampling setup are schematically shown in Figure S12 with accompanying descriptions. The results revealed that CO_2_ (5 vol%, in Ar) was converted into a mixture of 4.4 vol% CO_2_, 0.6 vol% CO, and 0.3 vol% O_2_ (in Ar) by plasma, resulting in a 14% CO_2_ conversion. On the OCs, further reactions led to a gas composition of, for instance, 0.9 vol% CO_2_, 4.1 vol% CO, and 0.0 vol% O_2_ (in Ar) in the case of Ce_0.7_Zr_0.3_O_2−δ_, achieving a total CO_2_ conversion of up to 84%.

To understand whether the role of plasma in PCLCS is exclusively the production of a CO_2_/CO/O_2_/O gas mixture, which is potentially more efficiently reduced on subsequent OCs compared to pure CO_2_, we conducted additional thermal CL tests employing the intermediate gas composition (4.4 vol% CO_2_, 0.6 vol% CO, 0.3 vol% O_2_, in Ar) in a tube furnace over reduced Ce_0.7_Zr_0.3_O_2−δ_ at the same temperature of 320 °C as the PCLCS system. The time-resolved gas composition results, depicted in Figure S13, indicate effective O_2_ capture by the reduced Ce_0.7_Zr_0.3_O_2−δ_ at this temperature. However, no conversion of CO_2_ to CO is observed over the OC for the intermediate gas composition, suggesting the presence of additional synergistic effects between the plasma and the OC.

Plasma has proven the ability of modifying the physicochemical properties of catalysts, influencing the kinetics of chemical reactions, as previously reported.^12,63^ To investigate whether such modifications occur in the context of PCLCS, the impact of plasma treatment (Ar as the carrier gas) on the chemical status of reduced Ce_0.7_Zr_0.3_O_2_ was evaluated through XPS analysis. The results presented in Figure S14 indicate negligible changes in the surface valence states of Ce_0.7_Zr_0.3_O_2_ before and after plasma treatment. This observation aligns logically with the experimental setup, where a distance of 2 mm between the OCs and the plasma in the PCLCS system in principle contributes to negligible alterations in the surface chemistry.

The above findings imply that the synergy between plasma and OC in PCLCS must be linked to the plasma composition and is likely attributed to the generation of excited CO_2_* molecules within the plasma, which subsequently undergo further splitting on the OCs to yield CO. The fraction of electron energy transferred to different channels of CO_2_ excitation and ionization in plasma was thus calculated, as a function of the reduced electric field (*E/n*) for the investigated 95 vol% Ar + 5 vol% CO_2_ gas mixture, from the corresponding cross-sections of the electron impact reactions, by using the electron Boltzmann equation solver BOLSIG^+^.^64^ The results, presented in Figure S15, suggest the abundance of both electronically and vibrationally excited CO_2_* molecules in the RGA plasma with an *E/n* range of 21–25 Td.

For CeO_2_-based OCs, oxygen vacancies are recognized as potent surface sites for the reactant adsorption and subsequent activation. Moreover, the formation of oxygen vacancies creates pathways for oxygen transport through the bulk lattice for surface reactions.^54^ In a typical CO_2_ splitting reaction over OC, CO_2_ molecules are adsorbed on the sites of either surface vacancies and activated into carbonate intermediates. Simultaneously, carbonates undergo rapid splitting into CO and O atoms, which either exchange with oxygen vacancies on the surface or migrate into the bulk crystal to recharge the Ce^3+^ cations in the OCs, benefiting from excellent mobility of lattice oxygen.^57^

**Figure 5:**
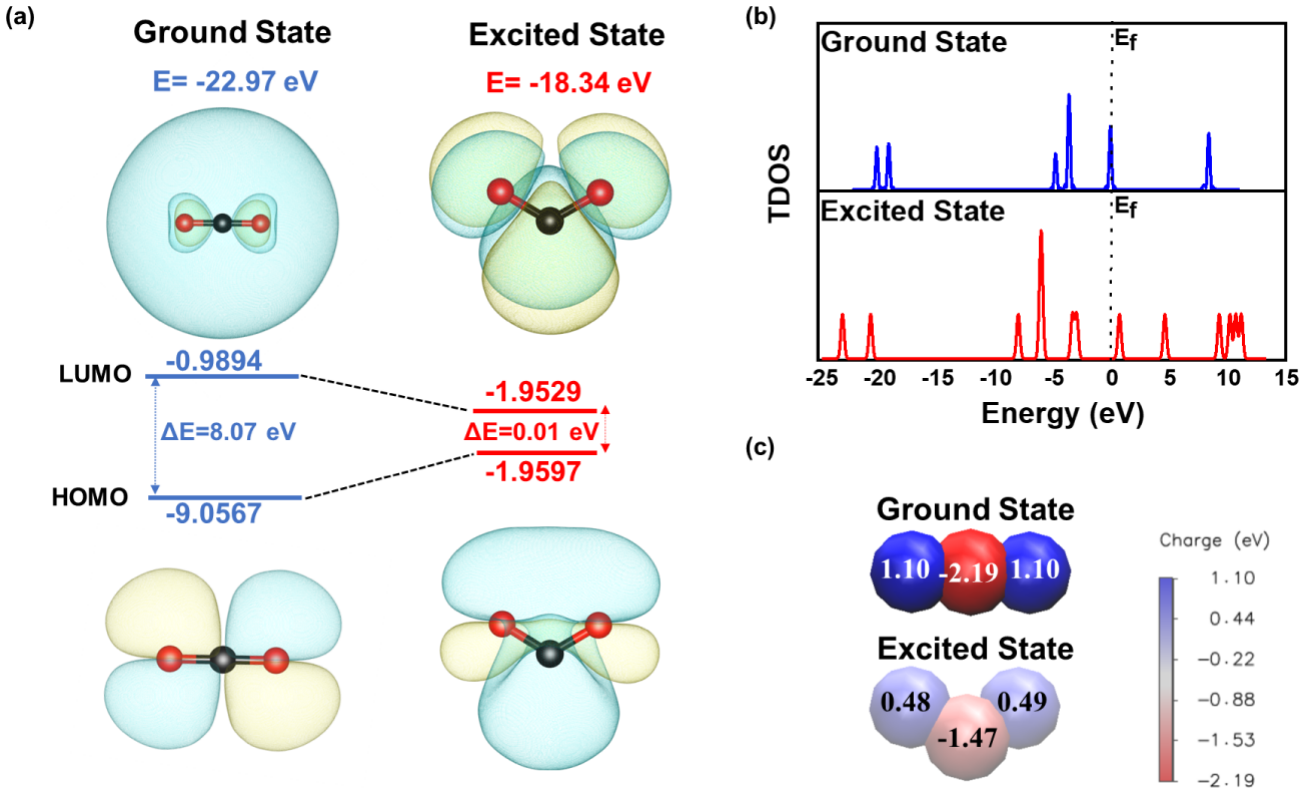
Elucidating different states of CO_2_. (a) HOMO and LUMO of CO_2_ in the ground state and excited state, (b) TDOS of CO_2_ in the ground state and excited state, and (c) Bader charge analysis of CO_2_ in the ground state and excited state.

DFT calculations were further conducted to elucidate the difference between excited and ground state CO_2_ over reduced Ce_0.7_Zr_0.3_O_2−δ_ in the above process. The electronic state of CO_2_ excitation in the gas phase was constructed by electron relocation from the highest occupied molecular orbital (HOMO) to the lowest unoccupied molecular orbital (LUMO) of the ground-state CO_2_.^65^ As illustrated in Figure 5a, after fixing the occupation of the electronic state, a structural distortion with a bending geometry occurs, along with a total energy increase of −22.97 eV toward −18.34 eV, signifying a less stable molecular structure. It should be noted that a similar bending excited state CO_2_ was also reported experimentally.^66^ Simultaneously, the LUMO energy significantly decreased from −9.06 eV to −1.96 eV and the HOMO–LUMO gap shrank from −8.07 eV to −0.01 eV before and after excitation. This makes it easier for the frontier electron to transfer from the occupied antibonding orbital σ* to the unoccupied π* orbit, indicating that the molecule tends to be easily activated during the reaction process.^67^

The total density of states (TDOS) of the ground-state and excited-state CO_2_, shown in Figure 5b, confirms this phenomenon: the LUMO of the excited state splits into two, resulting in the occupation of lower energy nondegenerate orbits.^68^ The Bader charge analysis, presented in Figure 5c, shows that a −0.50 eV charge is transferred to the vacuum charge after excitation, reconfirming a more active intermediate CO_2_ that reacts over Ce_0.7_Zr_0.3_O_2−δ_.

**Figure 6:**
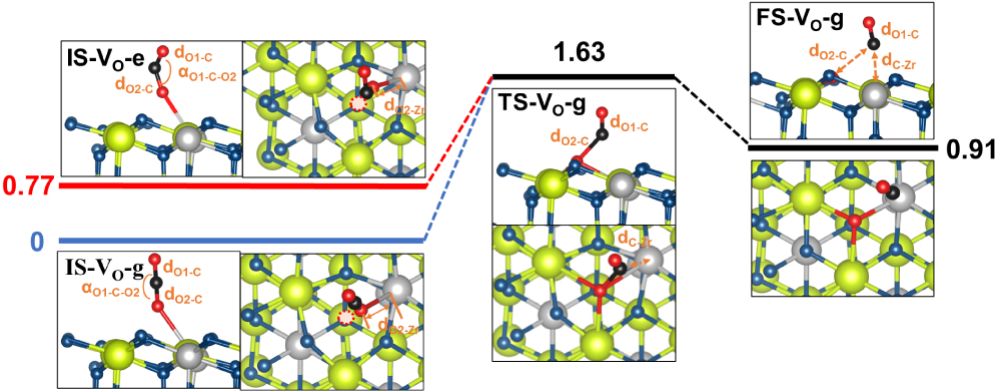
DFT calculations of different states of CO_2_ splitting on Ce_0.7_Zr_0.3_O_2−δ_. Potential energy pathway of CO_2_* → CO+O on Ce_0.7_Zr_0.3_O_2−δ_ (111) surface, where “e” represents the excited-state CO_2_* and “g” denotes the ground-state CO_2_.

The potential energy pathway of CO_2_ splitting on the Ce_0.7_Zr_0.3_O_2−δ_ (111) surface is presented in Figure 6, and more details are shown in Section S2.3. The calculation results show that the reaction involving excited CO_2_* exhibits a considerably lower activation barrier (0.86 eV), almost half of that associated with the ground state of CO_2_ (1.63 eV). Additionally, the reaction energy drops remarkably from 0.91 to 0.14 eV. These findings suggest that plasma-excited CO_2_* is significantly more reactive over the oxygen vacancies of Ce_0.7_Zr_0.3_O_2−δ_ compared to the ground-state CO_2_ for splitting reactions.

**Figure 7:**
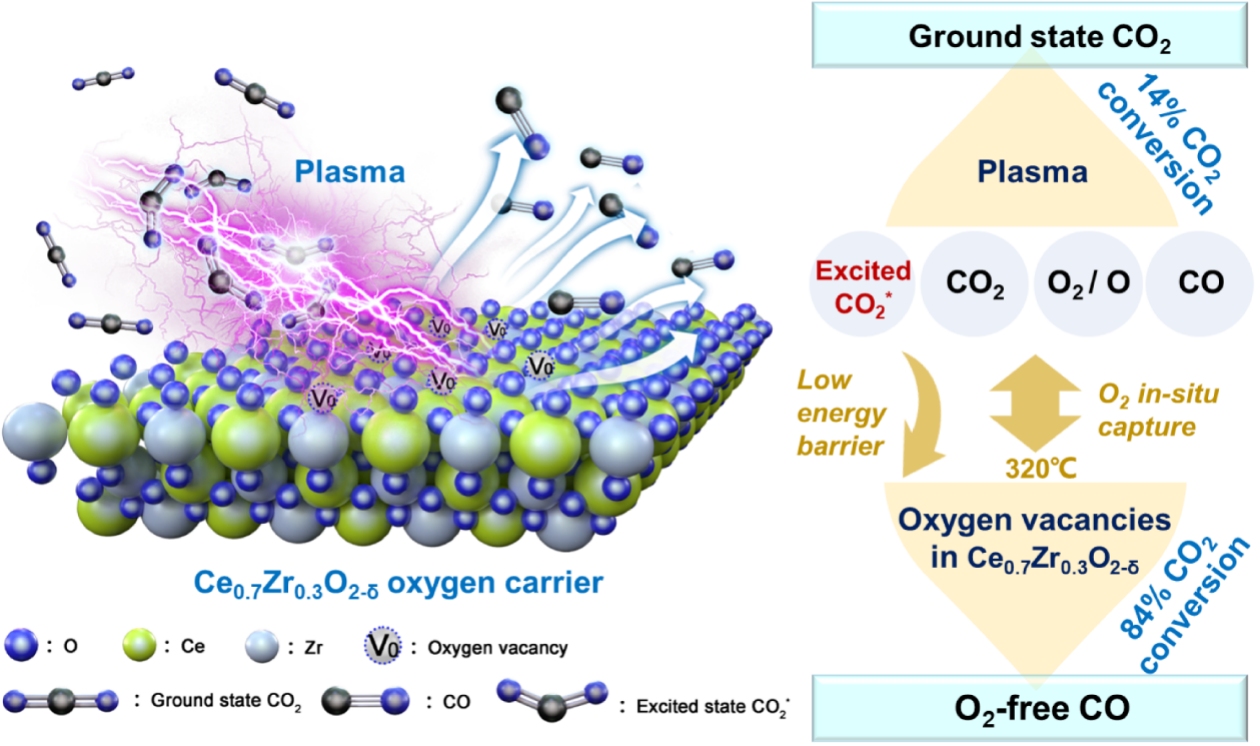
Schematic of the synergy between plasma and Ce_0.7_Zr_0.3_O_2−δ_ in the PCLCS.

The above investigation and analyses enable us to propose the underlying mechanism of the synergy between plasma and OCs, as schematically shown in Figure 7. 1) Plasma rapidly elevates the mixture temperature through energy transfer from electrons to neutral molecules, providing a temperature of around 320 °C for the thermal reactions over the OCs, eliminating the need for an external heat source. 2) Plasma initiates the activation and partial decomposition of the stable CO_2_ molecules, facilitated by energetic electrons and the generated reactive species. This process yields a mixture of CO, O_2_, or O and electronically/vibrationally excited CO_2_* molecules (besides ground-state CO_2_), contributing to a 14% conversion of CO_2_. 3) The excited CO_2_* then efficiently undergoes splitting over the Ce_0.7_Zr_0.3_O_2−δ_ OC, which possesses abundant oxygen vacancies, exhibiting a substantially reduced activation barrier compared to the ground-state CO_2_. Note that this process was proven to be unfeasible for the ground-state CO_2_ at 320 °C. 4) The O_2_ or O produced by plasma is captured in situ by the oxygen vacancies in Ce_0.7_Zr_0.3_O_2−δ_. We also compared the adsorption energies of O_2_/O and CO_2_ on the Ce_0.75_Zr_0.25_O_2−δ_ (111) surface, the results of which are summarized in Table S8. Both O_2_ and O exhibit higher adsorption energies compared to CO_2_. Therefore, there may be competition between O_2_/O and CO_2_ for oxygen vacancies in the OCs. However, given the relatively low concentration of O_2_/O compared to that of CO_2_ in the system (e.g., 4.4% CO_2_ and 0.3 vol% O_2_ after plasma, as measured), the influence of the concentration of O_2_/O is expected to be unimportant. This not only significantly contributes to the formation of O_2_-free CO but also facilitates the forward progression of the CO_2_ decomposition reaction, adhering to Le Chatelier’s principle.

The synergistic interaction between plasma and Ce_0.7_Zr_0.3_O_2−δ_ in the PCLCS system achieves a CO_2_ conversion of up to 84%, producing the desired O_2_-free CO product at a fairly low temperature of 320 °C. Last but not least, plasma can be powered by renewable energy sources and operates in an intermittent and decentralized manner. As a turnkey process, it allows for instant on/off switching and has no economy of scale.

## Conclusions

In this study, we introduce a novel PCLCS system that integrates an RGA plasma with a Ce_*x*_Zr_1–*x*_O_2_ OC, enabling efficient conversion of CO_2_ into an O_2_-free CO at mild temperatures.

Incorporating Zr into CeO_2_ substantially enhances the reaction performance by forming a Ce–Zr–O solid solution due to improved oxygen vacancy formation and lattice oxygen mobility, as experimentally and theoretically evidenced. The PCLCS system utilizing Ce_0.7_Zr_0.3_O_2−δ_ achieves a high CO_2_ conversion of up to 84% and a CO yield of 1.3 mmol g^–1^, with no measurable O_2_. This exceptional performance is realized at a mild temperature of only 320 °C, highlighting the superiority of the proposed system over traditional thermal CL processes (typically at 650–1000 °C). Furthermore, the achieved conversion surpasses those reported in NTP-based CO_2_ splitting processes (only 4%–50%), indicating a robust synergy between plasma and Ce_0.7_Zr_0.3_O_2−δ_.

In PCLCS, plasma initially activates and partially decomposes the stable CO_2_ molecules to yield CO, O_2_/O, and electronically/vibrationally excited CO_2_*, contributing to a 14% conversion of CO_2_. Importantly, the excited CO_2_* can then efficiently decompose over Ce_0.7_Zr_0.3_O_2−δ_ that possesses abundant oxygen vacancies, exhibiting a substantially reduced activation barrier (0.86 eV) compared to that of the ground-state CO_2_ (1.63 eV), as confirmed by DFT calculations. The strong O_2_/O capture ability of Ce_0.7_Zr_0.3_O_2−δ_ further accelerates the CO_2_ decomposition reactions, facilitating the generation of O_2_-free CO.

The proposed PCLCS strategy, which can be powered by renewable electricity in an intermittent and decentralized manner due to the instant on/off switching feature of plasma, is poised to emerge as a viable solution for addressing the grand challenge of conversion of CO_2_ to clean CO.

## Methods

### Experimental Setup and Methods

The PCLCS setup comprising a custom-built RGA reactor and a quartz cover housing OCs is illustrated in Figure 1 in the manuscript. Detailed configuration of the setup and further information on the experimental system are presented in Figures S1 and S2, respectively. The RGA reactor is composed of a cone-shaped inner anode and a circular cathode. A direct current (DC) power source (Teslaman TLP2040) powered the discharge in series for stabilizing the discharge current. The reactant gas, a mixture of 5 vol% CO_2_ diluted with 95 vol% Ar, entered through three tangential inlets at the bottom of the reactor, inducing a swirling flow. The total gas flow rate was controlled at 500 mL min^−1^ using mass flow controllers for both CO_2_ and Ar. An annular magnet situated outside the cathode generated an upward magnetic field. The interplay of swirling flow and Lorentz force caused the arc to ascend and finally rotate rapidly around the inner anode, forming a stable plasma “disc” conducive to chemical reactions. More details on the RGA reactor can be found in our previous work.^37,69^ Voltage and current signals were measured using Tektronix P6015A and Tektronix TCP303 probes, with waveforms recorded by a Tektronix DPO4034B oscilloscope. The discharge power during experiments was then determined to range from 67.6 to 71.5 W.

As illustrated in Figure 1, a downstream quartz cover (inner diameter: 14 mm) housed the OCs for the second-stage reactions. Each experiment utilized 1 g of 20–40 mesh reduced pellet OCs, positioned approximately 2 mm from the plasma area. The length of the OC bed is approximately 0.6 mm. Note that no additional heating was applied to the reaction area. A thermocouple (type NR-81530K) was positioned in the proximity of the surface of the oxygen carriers to measure the reaction temperature. Before introducing the reactant gas, pure N_2_ (99.99%) purged the reactor until the reaction area reached room temperature.

A schematic of the experimental setup is presented in Figure S2. The setup consists of a gas feeding system, a power supply, an RGA reactor with a gas sampling set, and an oscilloscope with high-voltage and current probes for electrical parameter measurement. Effluent gases were continuously monitored using an NDIR gas analyzer (GASBOARD-3100, Wuhan Cubic Optoelectronic Co., Ltd.) and a gas chromatograph (Agilent 7890) equipped with a thermal conductivity detector (TCD) and two capillary columns (HP-PLOT 5A, HP-PLOT-Q).

Plasma CO_2_ splitting reaction. The reduced OCs with a size of 20–40 mesh were placed into the plasma reactor (a quartz tube with 14 mm inside diameter), and then, the CO_2_ (5 vol% CO_2_/Ar) flowed through the reactor for reacting with the OCs. The outlet gases were monitored by an NDIR gas analyzer (GASBOARD-3100, Wuhan Cubic Optoelectronic Co., Ltd.).

Successive PCLCS testing. After the Ce_*x*_Zr_1–*x*_O_2_ OC is reduced by H_2_ for 1 h, pure N_2_ (99.99%) was introduced to purge the reactor instead of the H_2_ until the reactor concentration drops to room temperature. Then, the reduced OCs were placed into the plasma reactor for the plasma CO_2_ splitting reaction. The outlet gases were monitored by an NDIR gas analyzer (GASBOARD-3100, Wuhan cubic optoelectronic Co., Ltd.).

The effectiveness of PCLCS was evaluated based on CO_2_ conversion, CO yield, CO purity in the gas products, and energy efficiency, with calculations performed using the following formulas:CO_2_ conversion:
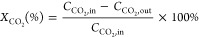
1CO yield:
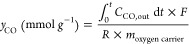
2CO purity in the gas products:
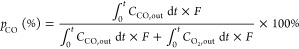
3energy efficiency:
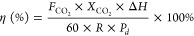
4where *C* represents the gas concentration, vol%; *t* signifies time, s; *F* denotes the gas flow rate, mL min^–1^; *P*_*d*_ is the plasma discharge power, W; Δ*H* is the reaction enthalpy of pure CO_2_ splitting, 280 kJ mol^–1^, and *R* corresponds to the volume of an ideal gas at standard temperature and pressure, 22.4 L mol^–1^. The value of *P*_*d*_ is 69 W in this work.

### Synthesis and Reduction of Ce_*x*_Zr_1–*x*_O_2_ Oxygen Carriers

In chemical looping processes, an OC with a high oxygen-carrying capacity is essential for facilitating the transport of energy and oxygen during redox reactions. Cerium-based oxides, such as CeO_2_, are recognized for high oxygen anion (O^2–^) conductivity, making them promising redox OCs.^47^ However, maintaining satisfactory O^2–^ conductivity and redox kinetics at relatively low temperatures (e.g., < 800 °C) remains challenging for unmodified CeO_2_.^20^ The concentration of oxygen vacancies and their energy barrier for migration become crucial at low temperatures, influencing the rate at which lattice oxygen participates in the redox reactions. The fluorite structure, however, provides an avenue to enhance O^2–^ conductivity and/or structural stability by accommodating cation dopants. Among various cations, the tetravalent Zr ion emerges as a suitable candidate due to its size similarity to Ce^4+^.^61^

In this study, Ce_1–*x*_Zr_*x*_O_2_ OCs, where *x* = 0.1, 0.2, 0.3, 0.4, and 0.5 in mole fraction, were synthesized for PCLCS experiments, by using the coprecipitation method. In a typical procedure, Ce(NO_3_)_3_·6H_2_O and Zr(NO_3_)_4_·5H_2_O were dissolved in deionized water, yielding a total cation concentration of 2.0 mol L^–1^. Subsequently, an 8 wt % ammonia aqueous solution was added dropwise to the mixed solution under continuous stirring until the pH reached 10. The resulting solid–liquid mixture underwent additional stirring for 3 h. After aging at room temperature for 12 h, the mixture was filtered and washed multiple times. The precipitate was subsequently dried at 110 °C for 24 h and calcinated at 800 °C for 2 h to obtain Ce_*x*_Zr_1–*x*_O_2_ powder OCs. Finally, the powder OCs were compacted at 10 MPa for 15 min, crushed, and sieved to produce pellet OCs with a particle size ranging from 20 to 40 mesh. The fresh OCs and the oxidized OCs after each PCLCS experiment (Ce_1–*x*_Zr_*x*_O_2_) were reverted to reduced Ce_1–*x*_Zr_*x*_O_2−δ_ in another half cycle using H_2_ in a heated fixed-bed reactor at 1 atm. The process involved initial heating in pure N_2_ at a temperature ramp of 10 °C min^–1^ from room temperature to 800 °C, followed by reduction under H_2_ (10 vol% H_2_/N_2_) for 1 h.

### Material Characterizations

Powder XRD patterns were acquired to investigate the crystallographic phase of OCs using a MiniFlex600 Rigaku XRD meter with Cu Kα radiation (λ= 0.15406 nm). The diffraction patterns were collected under ambient conditions within a 2θ range of 10°–90° with a step size of 2° min^–1^.

SEM (VERSA 3D, FEI) was employed to examine the morphology of the OCs. The as-prepared samples were sputter coated with a thin layer of gold, and imaging was performed at an electron beam acceleration of 3 kV.

The specific surface area of the OCs was determined using the BET method with a Quantachrome NOVA 2000e instrument, employing volumetric nitrogen adsorption at −196 °C.

TEM was conducted by using a Tecnai G^2^ TF30 S-Twin microscope operating at 300 kV. The specimens were crushed into a powder and then immersed in a small volume of ethanol. After sonicating the mixture for 10 min, a droplet of the suspension was allowed to dry on a holey carbon/Formvar-coated copper TEM grid.

XPS experiments were carried out using a Thermo Fisher Scientific K-Alpha^+^ system equipped with a monochromatic Al-Ka X-rays source. Spectra were recorded under sample purging at ambient temperature in a vacuum (residual pressure of <10^–7^ Pa). An electron flood gun compensated for sample charging during the measurement. The C 1 s signal at 284.8 eV served as an internal standard for calibration of the XPS signals.

H_2_-TPR was performed using a Quantanchrome Instrument. After standard cleaning pretreatment, 100 mg of OCs in a U-tube reactor was heated from room temperature to 900 °C with a heating rate of 10 °C min^–1^ in a 10 vol% H_2_/Ar flow (25 mL min^–1^).

CO_2_-TPO experiments were carried out on a microreactor system (Hiden Analytical Co.). Fresh samples were reduced in a 10 vol% CO_2_/Ar stream at 450 °C for 2 h, cooled to room temperature in pure Ar, and subjected to CO_2_ at room temperature for 1 h. Subsequently, the temperature was increased to 600 °C in flowing Ar. Gas compositions were analyzed using online mass spectrometer. Blank measurements on the OCs were also performed to identify contributions for carbonate species present on the OCs before and after CO_2_ exposure.

### DFT Calculations

Periodic energy calculations were conducted using the DFT approach, implemented in the Vienna Ab-intio Simulation Package (VASP) code.^70^ The generalized gradient approximation (GGA) with the Perdew–Burke–Ernzerhof (PBE) equation was adopted to calculate the exchange correlation energy, with core electrons (Ce: 5p^6^ 5d^1^ 4f^1^ 6s^2;^ Zr: 4s^2^ 4p^6^ 5s^2^ 5d^2^; O: 2s^2^ 2p^4^) treated using the projected augmented wave (PAW) approximation. The Kohn–Sham equations were solved with a plane wave cutoff energy of 400 eV and a 4 × 4 × 1 k-point grid supported by the Monkhorst–Pack Method, ensuring geometry optimization reached a force convergence threshold lower than 0.02 eV. More details about the selection of the computational parameters could be found in our previous work.^71^ All the systems were treated with the DFT+U methodology with a Hubbard parameter “U” of 5.00 eV to well describe the Ce(4f) electrons.^72^ Moreover, transition states (TS) involving O-vacancy immigration and the CO_2_ dissolution process were identified using a combined method of CI-NEB+DIMER.^73^ TS with a single vibrational frequency were emphasized.

The optimization of excited state CO_2_* adsorption involved fixing CO_2_* at its equilibrium position, while the Ce_0.75_Zr_0.25_O_2−δ_ bulk substrate underwent relaxation. Conversely, for ground-state adsorbate systems, structural relaxation was applied in all cases. The adsorption energy is defined as
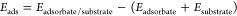
5

Herein, *E*_ads_ represents the adsorption energy of the intermediate in eV; *E*_adsorbate/substrate_ denotes the total energy of the entire adsorption system; *E*_adsorbate_ signifies the energy of the adsorbed molecules in the free state vacuum; and *E*_substrate_ refers to the energy of the surface system.

The reaction energy *E*_r_ and the activation barrier *E*_b_ are defined as
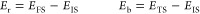
6where *E*_FS_, *E*_IS_, and *E*_TS_ refer to the total energy of the final state, initial state, and transition state, respectively.

## Supporting Information Available

Additional information on the methods and results of experiments and DFT calculations; summary of the PCLCS experimental results over reduced Ce_0.7_Zr_0.3_O_2−δ_ OCs (Table S1); fresh CeO_2_ and Ce_0.7_Zr_0.3_O_2_ lattice parameters and refinement factors (Table S2); computed formation energy of each oxygen vacancy in Ce_0.75_Zr_0.25_O_2−δ_ (Table S3); computed migration barrier of each oxygen migration to O_2_ in Ce_0.75_Zr_0.25_O_2−δ_ (Table S4); computed formation energy of each oxygen vacancy in Ce_0.75_Zr_0.25_O_2−δ_ (Table S5); computed migration barrier of each oxygen migration to O_2_ in Ce_0.75_Zr_0.25_O_2−δ_ (Table S6); computed migration barrier of each oxygen migration to O_2_ in Ce_0.75_Zr_0.25_O_2−δ_ (Table S7); computed adsorption energy of intermediates on Ce_0.7_Zr_0.3_O_2_ (111) surface (Table S8) (PDF)
