# Supplementary material for: Plasma Chemical Looping: Unlocking High-Efficiency CO2 Conversion to Clean CO at Mild Temperatures
Source: JACS Au. 2024 May 8;4(7):2462–73. doi: 10.1021/jacsau.4c00153 (PMC11267539; doi:10.1021/jacsau.4c00153)
Supplement: Supplementary file 1 — au4c00153_si_001.pdf [file au4c00153_si_001.pdf]

Supplemental Information

Plasma Chemical Looping: Unlocking High-Efficiency CO_2 Conversion to Clean CO at Mild Temperatures

Yanhui Long[#], Xingzi Wang[#], Hai Zhang[*], Kaiyi Wang, Wee-Liat Ong, Annemie Bogaerts, Kongzhai Li, Chunqiang Lu, Xiaodong Li, Jianhua Yan[*], Xin Tu[*], Hao Zhang[*]

[1]State Key Laboratory of Clean Energy Utilization, Zhejiang University, Hangzhou 310027, China.

[2]School of Mechanical Engineering, Shanghai Jiao Tong University, Shanghai 200240, China.

[3]College of Energy Engineering, ZJU-UIUC, Zhejiang University, Hangzhou 310027, China.

[4]Research group PLASMANT, Department of Chemistry, University of Antwerp, Universiteitsplein 1, Antwerp 2610, Belgium.

[5]State Key Laboratory of Complex Nonferrous Metal Resources Clean Utilization, Kunming University of Science and Technology, Kunming 650093, China.

[6]Department of Electrical Engineering and Electronics, University of Liverpool, Liverpool L69 3GJ, UK.

[7]Ningbo Innovation Center, Zhejiang University, Ningbo 315100, China.

[#] The two authors contribute equally to this work.

*Corresponding authors:

zhang_hao@zju.edu.cn; xin.tu@liverpool.ac.uk; zhanghai@sjtu.edu.cn; yanjh@zju.edu.cn

Table of contents

Section S1 Experiments 3

Figure S1. Schematic of the Plasma Chemical Looping CO_2 Splitting (PCLCS) setup.

Figure S2. Schematic of the experimental system.

Figure S3. Time-resolved concentrations of the gas products in PCLCS experiments over reduced $Ce_{1-x}Zr_xO_{2-\delta}$with different Zr content.

Figure S4. Time-resolved concentrations of the gas products in PCLCS experiments without OCs.

Figure S5. CO_2-TPO profile of the reduced $Ce_{0.7}Zr_{0.3}O_{2-\delta}$ oxygen carriers.

Figure S6. Redox stability of the PCLCS system over 10 cycles using reduced $Ce_{0.7}Zr_{0.3}O_{2-\delta}$: (a) CO yield and CO_2 conversion; (b) CO purity in the gas product.

Figure S7. (a) XRD patterns and (b) H_2-TPR profiles of fresh and cycled $Ce_{0.7}Zr_{0.3}O_2$. SEM images (1), TEM images (2), HRTEM images (3-4) and TEM-EDS (5-8) of (c) fresh and (d) cycled $Ce_{0.7}Zr_{0.3}O_2$.

Figure S8. SEM images of the fresh (a) and cycled (b) $Ce_{0.7}Zr_{0.3}O_2$ oxygen carriers.

Figure S9. XRD patterns of various OCs: (a) $Ce_{1-x}Zr_xO_2$ (x = 0, 0.1, 0.2, 0.3, 0.4, 0.5), (b) enlarged (111) reflections of CeO_2, and Rietveld refinement results of XRD patterns: (c) CeO_2, (d) $Ce_{0.7}Zr_{0.3}O_2$.

Figure S10. (a) H_2-TPR profiles and (b) cumulative H_2 uptake of the prepared $Ce_{1-x}Zr_xO_2$ OCs.

Figure S11. (a)O 1s and (b) Ce 3d XPS spectra of the fresh $Ce_{1-x}Zr_xO_2$ oxygen carriers with different Zr content.

Figure S12. Plasma in situ gas sampling device.

Figure S13. Time-resolved concentrations of the simulated plasma generated gas (4.4 vol% CO_2, 0.6 vol% CO, 0.3 vol% O_2 in Ar) over reduced $Ce_{0.7}Zr_{0.3}O_2$ at 320 °C in a tube furnace.

Figure S14. O 1s XPS spectra of reduced $Ce_{0.7}Zr_{0.3}O_2$ before and after Ar plasma treatment.

Figure S15. Fraction of electron energy transferred to different channels of CO_2 excitation and ionization, as a function of the reduced electric field (*E/n*).

Table S1. Summary of the PCLCS experimental results over reduced $Ce_{0.7}Zr_{0.3}O_{2-\delta}$ OCs.

Table S2. Fresh CeO_2 and $Ce_{0.7}Zr_{0.3}O_2$ lattice parameters and refinement factors.

Section S2 DFT Calculations 16

Figure S16. Model structures of (a) CeO_2 and (b) $Ce_{0.75}Zr_{0.25}O_2$.

Figure S17. Model Structures of (a) $Ce_{0.75}Zr_{0.25}O_2$ 2*2*2 supercell and (b) $Ce_{0.75}Zr_{0.25}O_{2-\delta}$ 2*2*2 supercell with Vo at O_2 site.

Figure S18. Energy potential profile along the O1→V_{O2}, O3→V_{O2} and O4→V_{O2} pathways in $Ce_{0.75}Zr_{0.25}O_{2-\delta}$ bulk.

Figure S19. Model Structures of (a) $Ce_{0.75}Zr_{0.25}O_2$ (111) surface, (b) $Ce_{0.75}Zr_{0.25}O_{2-\delta}$ (111) surface with Vo at O_C site.

Figure S20. Energy potential profile along the Oa→V_{O_C}, Ob→V_{O_C} and O_c→V_{O_C} pathways on $Ce_{0.75}Zr_{0.25}O_{2-\delta}$ (111) surface.

Table S3. Computed formation energy of each oxygen vacancy in $Ce_{0.75}Zr_{0.25}O_{2-\delta}$.

Table S4. Computed migration barrier of each oxygen migration to O_2 in $Ce_{0.75}Zr_{0.25}O_{2-\delta}$.

Table S5. Computed formation energy of each oxygen vacancy in $Ce_{0.75}Zr_{0.25}O_{2-\delta}$.

Table S6. Computed migration barrier of each oxygen migration to O_2 in $Ce_{0.75}Zr_{0.25}O_{2-\delta}$.

Table S7. Geometric Properties (explained in **Figure 6**) of ground state and excited state CO_2^* dissolution.

Table S8. Computed adsorption energy of intermediates on $Ce_{0.7}Zr_{0.3}O_2$ (111) surface.

Section 1. Experiments

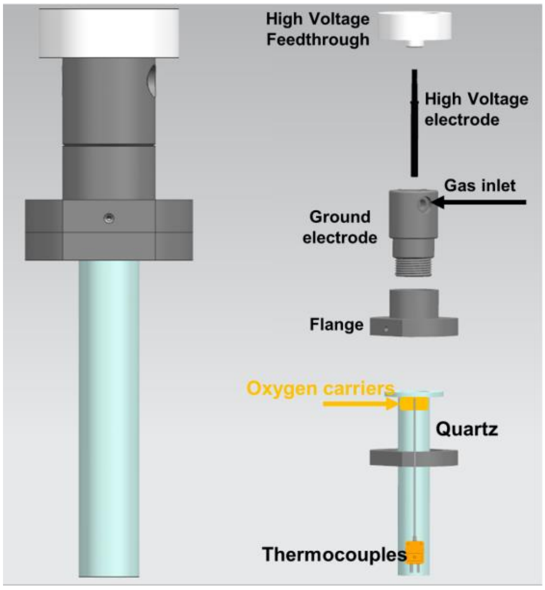

Figure S1. Schematic of the Plasma Chemical Looping CO_2 Splitting (PCLCS) setup.

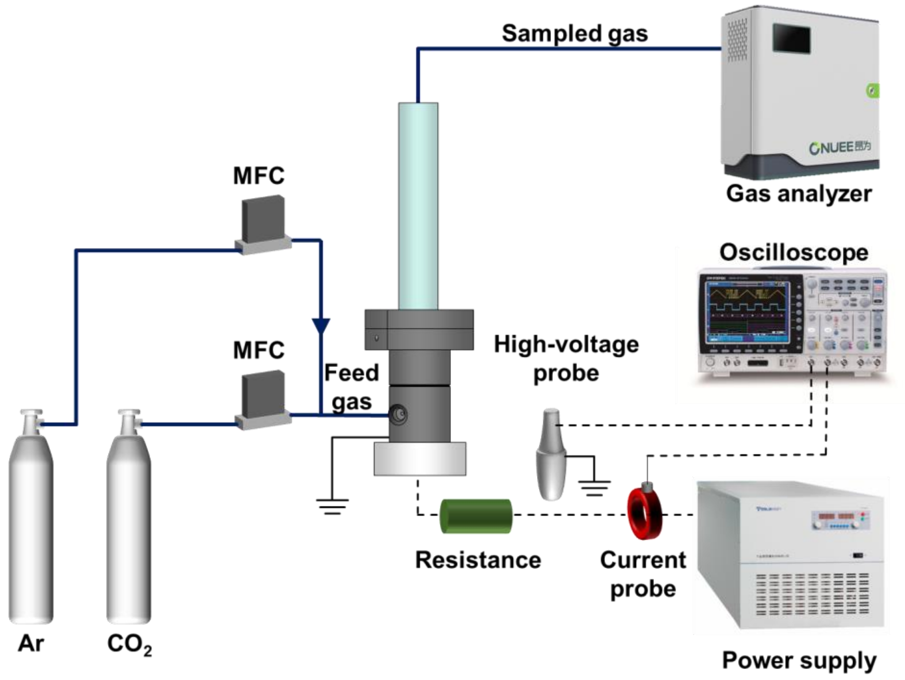

Figure S2. Schematic of the experimental system.

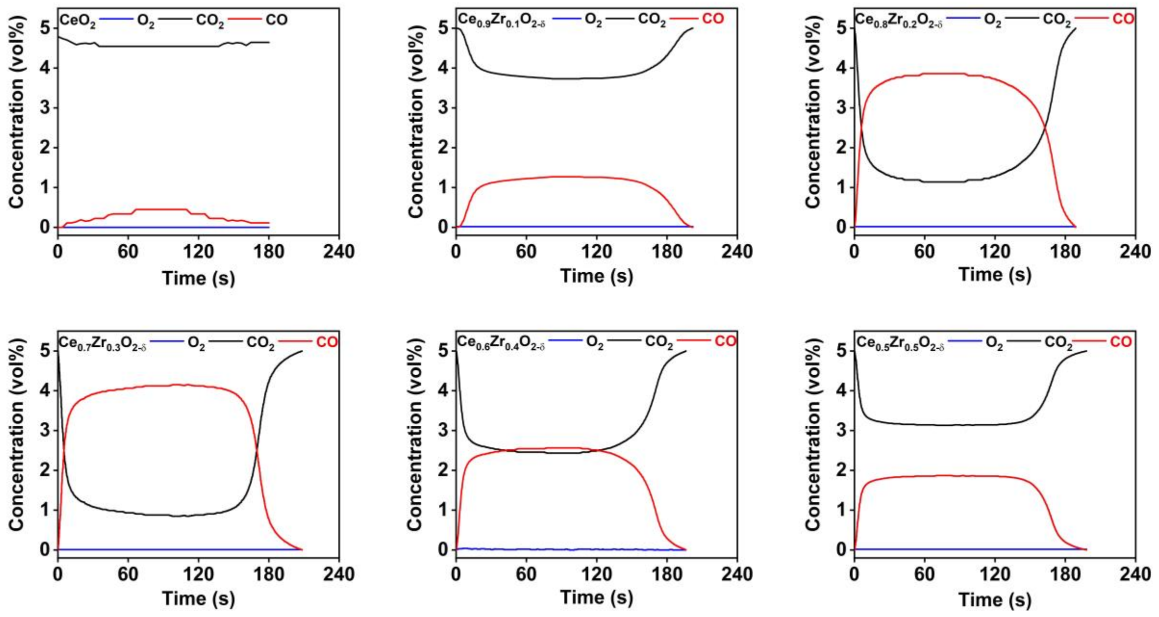

Figure S3. Time-resolved concentrations of the gas products in PCLCS experiments over reduced $Ce_{1-x}Zr_xO_{2-\delta}$ with different Zr contents.

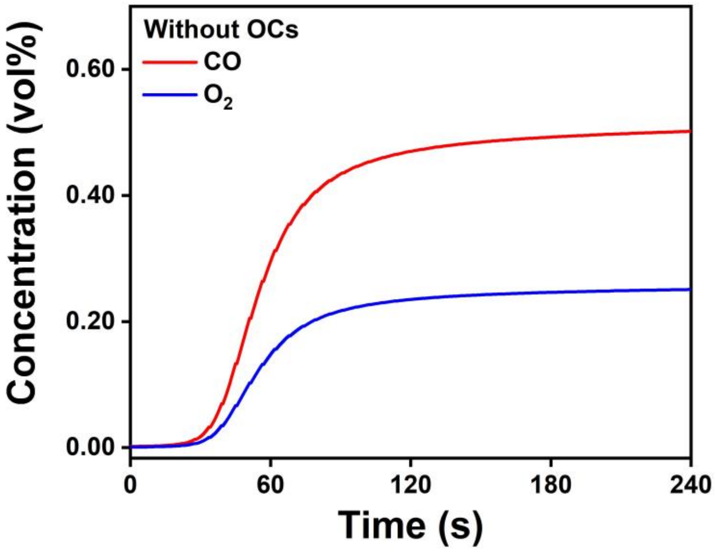

Figure S4. Time-resolved concentrations of the gas products in PCLCS experiments without OCs.

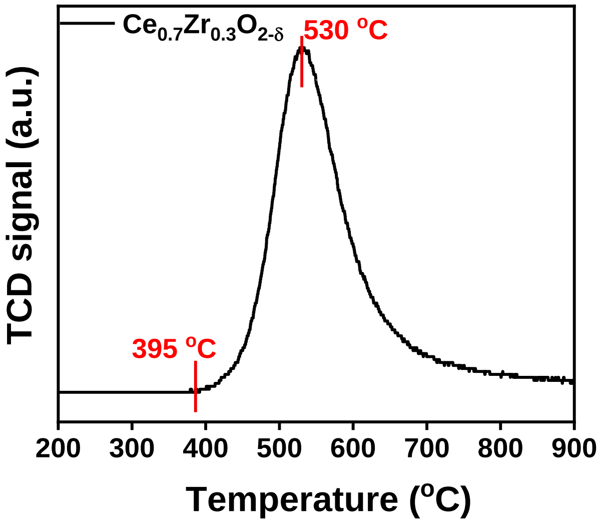

Figure S5. CO_2-TPO profile of the reduced $Ce_{0.7}Zr_{0.3}O_{2-\delta}$ oxygen carrier.

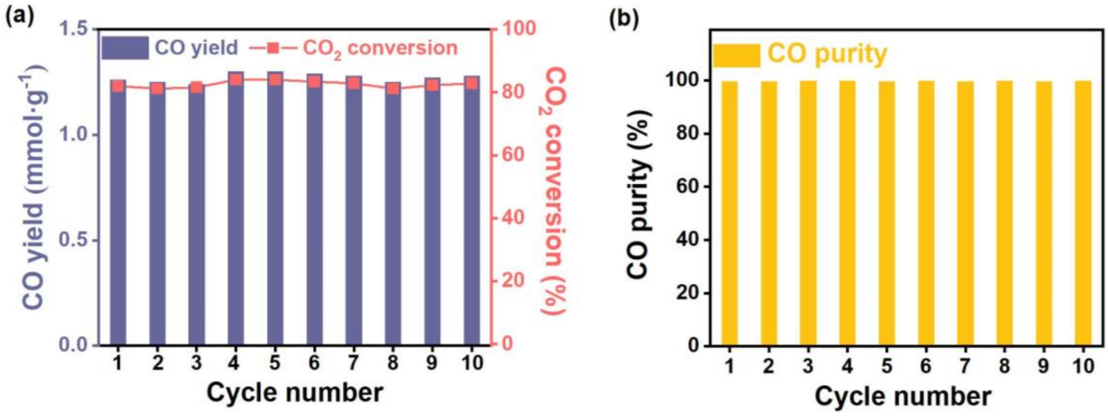

Figure S6. Redox stability of the PCLCS system over 10 cycles using reduced $Ce_{0.7}Zr_{0.3}O_{2-\delta}$: (a) CO yield and CO_2 conversion; (b) CO purity in the gas product.

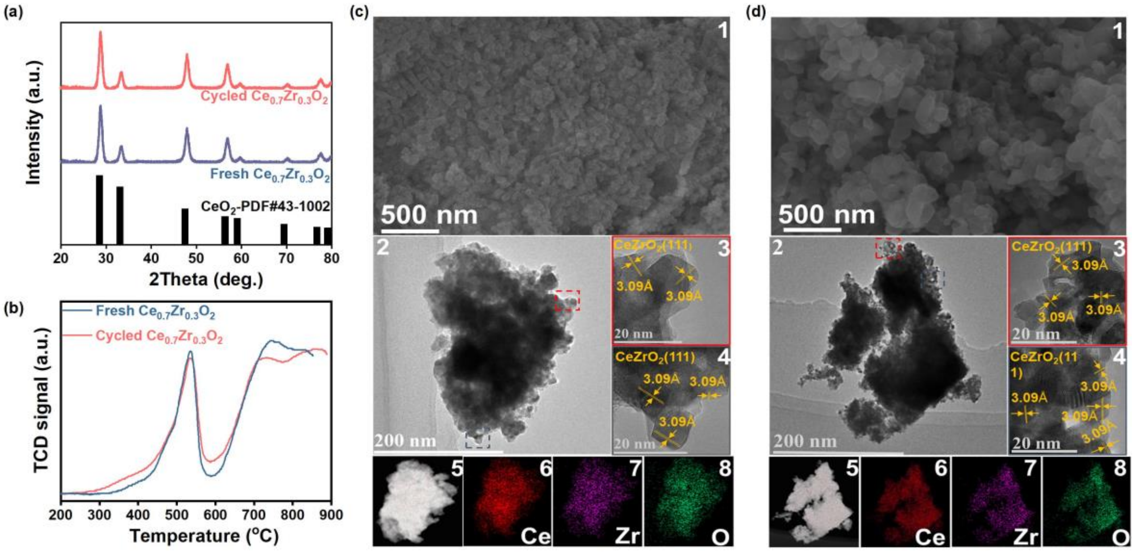

Figure S7. (a) XRD patterns and (b) H_2-TPR profiles of fresh and cycled $Ce_{0.7}Zr_{0.3}O_2$. SEM images (1), TEM images (2), HRTEM images (3-4) and TEM-EDS (5-8) of (c) fresh and (d) cycled $Ce_{0.7}Zr_{0.3}O_2$.

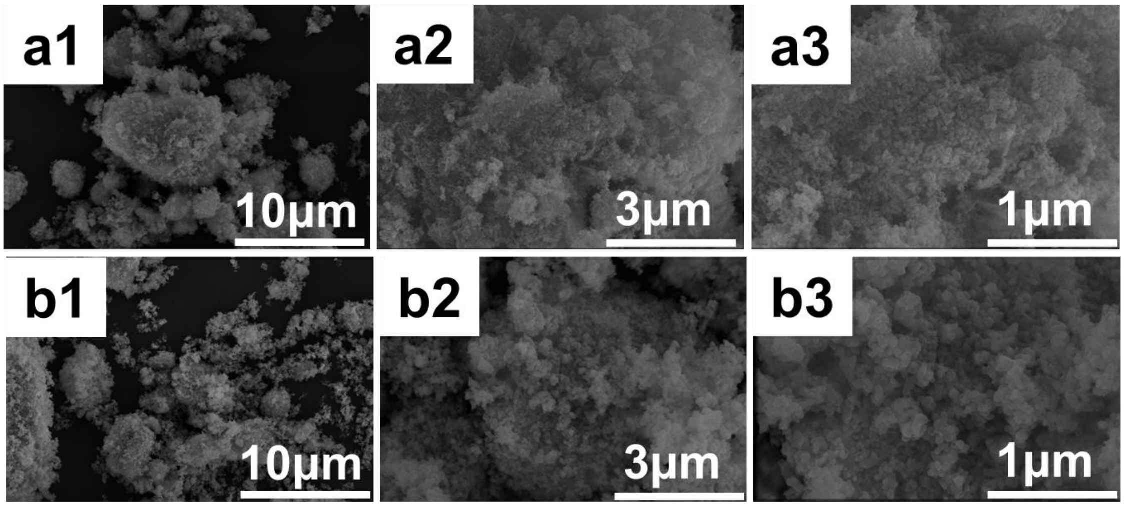

Figure S8. SEM images of the fresh (a) and cycled (b) $Ce_{0.7}Zr_{0.3}O_2$ oxygen carriers.

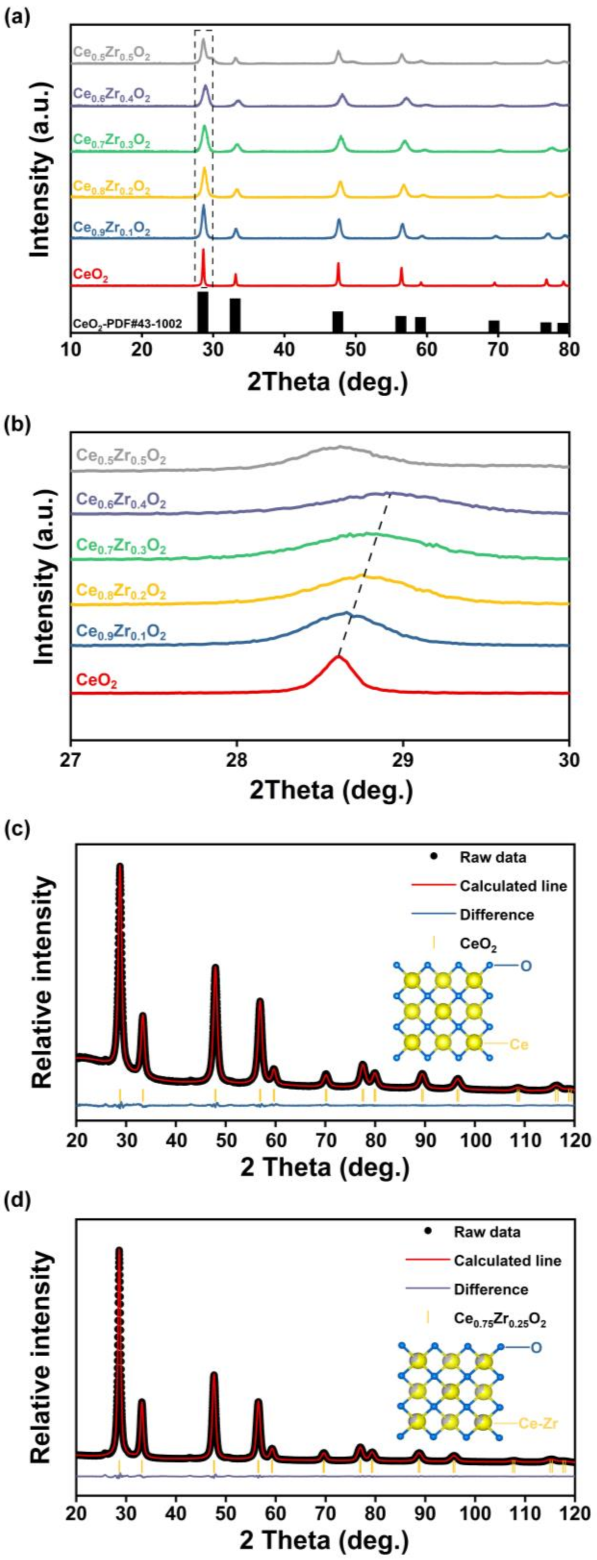

Figure S9. XRD patterns of various OCs: (a) $Ce_{1-x}Zr_xO_2$ (x = 0, 0.1, 0.2, 0.3, 0.4, 0.5), (b) enlarged (111) reflections of CeO_2, and Rietveld refinement results of XRD patterns: (c) CeO_2, (d) $Ce_{0.7}Zr_{0.3}O_2$.

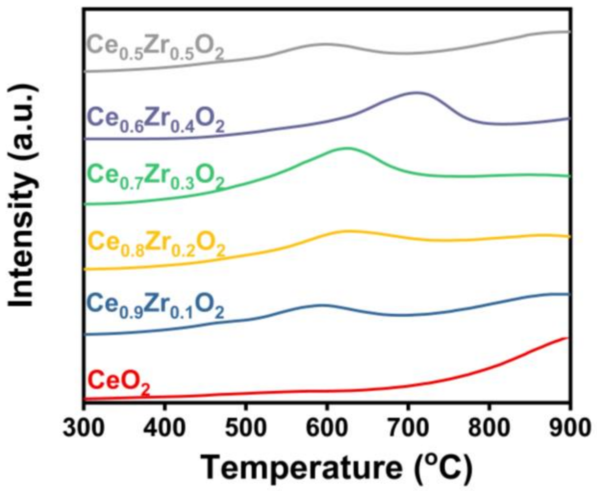

Figure S10. H_2-TPR profiles of the prepared $Ce_{1-x}Zr_xO_2$ OCs.

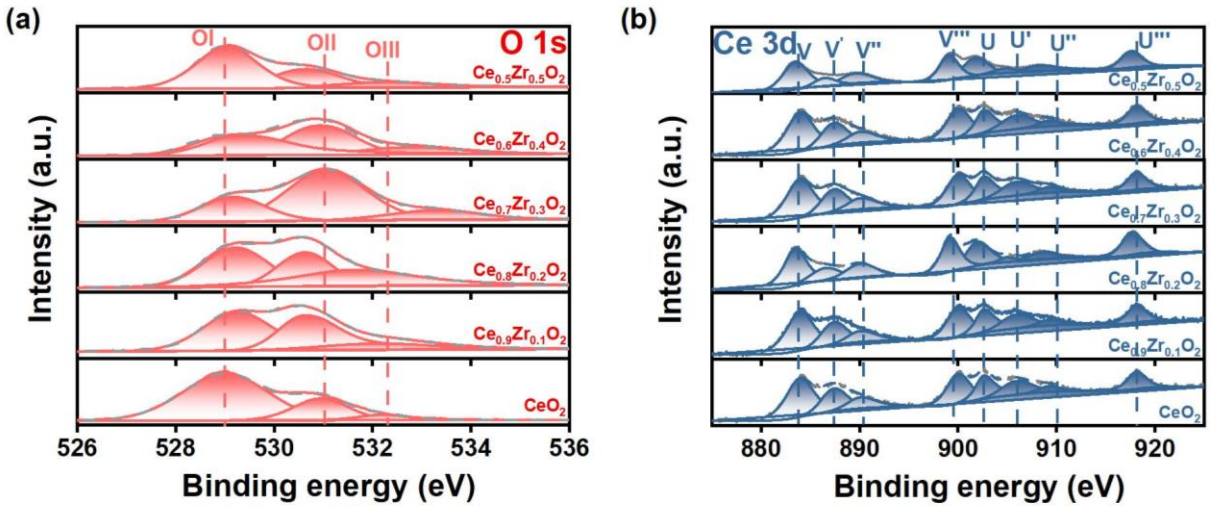

Figure S11. (a) O 1s and (b) Ce 3d XPS spectra of the fresh $Ce_{1-x}Zr_xO_2$ oxygen carriers with different Zr content.

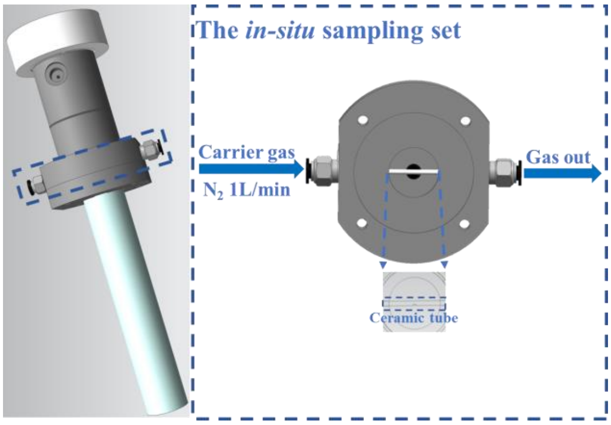

Figure S12. Plasma *in-situ* gas sampling device.

The *in-situ* sampling set was an alumina ceramic tube (inner / outer diameter=1 mm / 2 mm) with a hole of 0.4 mm in diameter in the middle. During the sampling, carrier gas N_2 with a flow rate of 1 L/min was injected into the tube, forming a fast flow (21 m/s) that can extract reactive gas from the chamber into the ceramic tube. In this way, the sampled gas can be *in-situ* diluted and cooled down, thereby “freezing” the chemical composition of the sampled gases by largely inhibiting the secondary reactions during sampling.

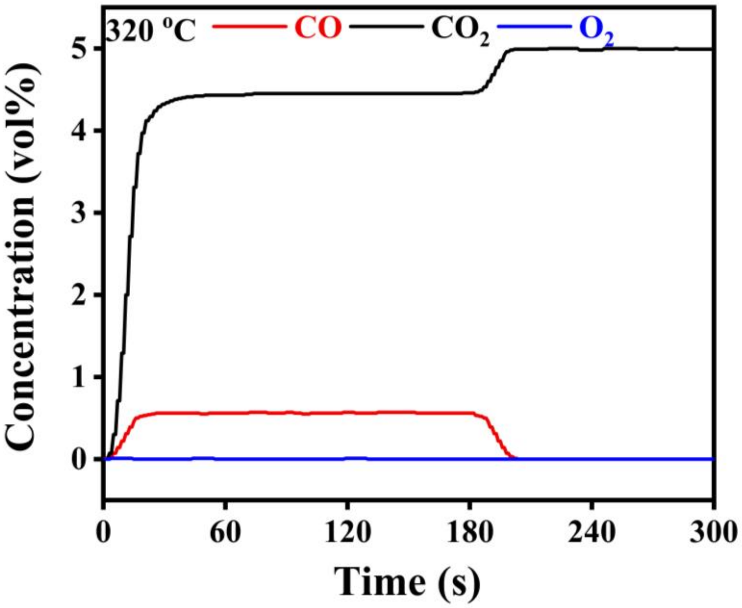

Figure S13. Time-resolved concentrations of the simulated plasma generated gas (4.4vol% CO_2, 0.6vol% CO, 0.3vol% O_2 in Ar) over reduced $Ce_{0.7}Zr_{0.3}O_2$ at 320 °C in a tube furnace.

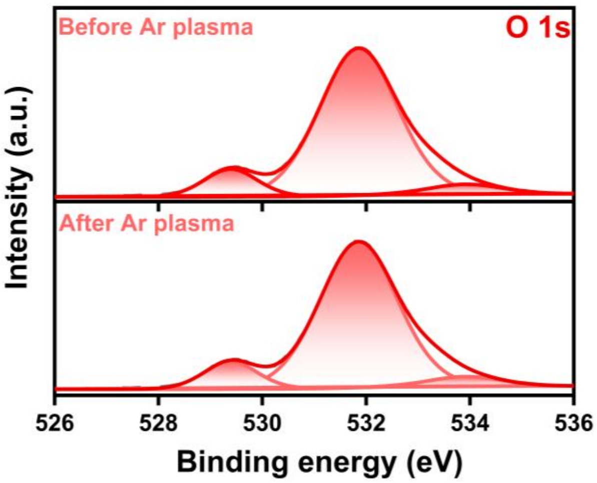

Figure S14. O 1s XPS spectra of reduced $Ce_{0.7}Zr_{0.3}O_2$ before and after Ar plasma treatment.

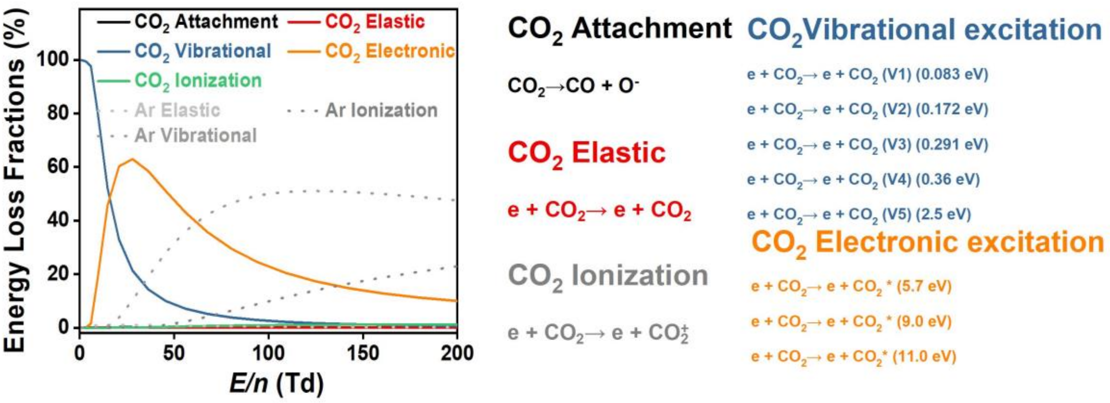

Figure S15. Fraction of electron energy transferred to different channels of CO_2 excitation and ionization, as a function of the reduced electric field (*E/n*). The cross-sections of the electron-impact reactions for the investigated 95vol% Ar + 5vol% CO_2 are obtained from[1]. The *E/n* of the RGA used in PCLCS is around 21-25 Td and is marked in the Figure S14.

The *E/n* was evaluated using the following formulas.

$$E/n = \frac{U \times T \times k_B}{d \times P} \times 10^{21} \tag{1}$$

where Td; *U* signifies reactor voltage, V; *d* denotes the discharge gap, m; and P represents atmospheric pressure, 1.01325×10^5 Pa; k_B corresponds to Boltzmann constant, 1.380649×10^{-23} J K^{-1}; *T* is the reaction temperature, K. The values of *V*, *d* and *T* used in this work are 290-292 V, 0.001 m and 593 K.

Table S1. Summary of the PCLCS experimental results over reduced $Ce_{0.7}Zr_{0.3}O_{2-\delta}$ OCs.

	1.0 g OCs[a]	1.5 g OCs[a]	2.0 g OCs[a]	1.0g OCs[b]
Discharge power (W)	69.0	68.7	69.0	69.0
CO yield (mmol)	1.2	1.8	2.2	1.3
CO_2 conversion (%)	7	8.9	12	84
Energy efficiency (%)	10.6	13.5	18.2	6.4

a: gas mixture: pure CO_2, 500sccm.

b: gas mixture: 5vol% CO_2+95vol% Ar, 500sccm.

Table S2. Fresh CeO_2 and $Ce_{0.7}Zr_{0.3}O_2$ lattice parameters and refinement factors.

Oxygen carriers	a (Å)	b (Å)	c (Å)	R_p (%)	R_{wp}(%)	χ^2
CeO_2	5.3674	5.3674	5.3674	2.07	1.54	2.352
$Ce_{0.7}Zr_{0.3}O_2$	5.4027	5.4027	5.4027	1.77	2.59	3.592

Section S2. DFT calculations

S2.1 Model construction, oxygen formation energy and migration barrier calculations of bulk CeO_2 and $Ce_{0.75}Zr_{0.25}O_{2-\delta}$

The atomic model of pristine CeO_2 unit cell is shown in Figure S15(a), the optimized lattice constant is 5.41 Å which is in accordance with the reported experimental 5.41 Å[2]. The $Ce_{0.75}Zr_{0.25}O_2$ unit cell is built by replacing eight Ce atoms by eight Zr atoms, as shown in Figure S15(b). Due to symmetry, the selection of the Zr doping in the CeO_2 unit cell is unique. Subsequently, a 2 × 2 × 2 supercell of $Ce_{0.75}Zr_{0.25}O_2$ bulk structure is built to avoid artificial interactions induced by the periodic boundary condition. In this model, all 4 non-equivalent oxygen sites depicted in Figure S16 are considered to calculate the oxygen vacancy formation energy ($E_{O\_Formation}$) (Table S3) and the most stable one (O_2 site) is chosen for subsequent calculations. The computed lowest $E_{O\_Formation}$ of $Ce_{0.75}Zr_{0.25}O_{2-\delta}$ (111) is 2.43 eV, which is much lower than pristine CeO_2 (2.99 eV). Upon the formation of one V_O, all adjacent 3 O atoms are considered to migrate to the vacancy site, and the corresponding reaction energies are listed in the Table S4. Then the transition state calculations of the migration these oxygen atoms are performed as shown in Figure S17. The computed lowest migration barrier (0.34 eV) is lower than it is in CeO_2 (0.46 eV), suggesting that the oxygen migration is more likely to occur in $Ce_{0.75}Zr_{0.25}O_{2-\delta}$ than in CeO_2. It is found that the O atom initially neighboring to three oxygen vacancies is most likely to migrate (O1 → V_{O2}).

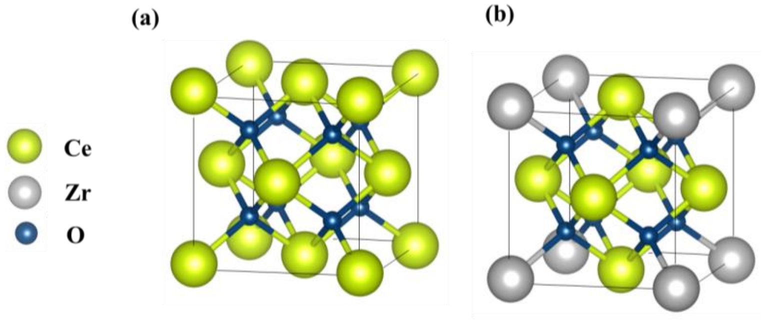

Figure S16. Model structures of (a) CeO_2 and (b) $Ce_{0.75}Zr_{0.25}O_2$.

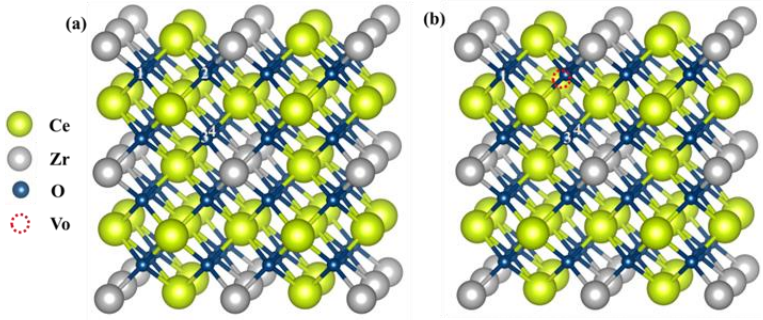

Figure S17. Model Structures of (a) $Ce_{0.75}Zr_{0.25}O_2$ 2*2*2 supercell and (b) $Ce_{0.75}Zr_{0.25}O_{2-\delta}$ 2×2×2 supercell with Vo at O_2 site.

Table S3. Computed formation energy of each oxygen vacancy in $Ce_{0.75}Zr_{0.25}O_{2-\delta}$ ($E_{O\_Formation} = E_{substrate} - E_{substrate\_vo} + E_O$, where $E_{substrate}$, $E_{substrate\_vo}$ and E_O represents the total energy of the entire system, the total energy of the system with O vacancy and the energy of O in free state vacuum).

**Vacancy Site**	$\mathbf{E_{O\_Formation}}$ **(eV)**
O1	2.44
O2	2.43
O3	2.44
O4	2.44

Table S4. Computed migration barrier of each oxygen migration to O_2 in $Ce_{0.75}Zr_{0.25}O_{2-\delta}$ (Migration barrier E_m: same as the activation barrier (general) calculated $E_m = E_{TS} - E_{IS}$, where E_{TS} indicates the total energy of the transition state).

**Migration pathway**	$\mathbf{E_m}$ **(eV)**
O1→V_{O2}	0.34
O3→V_{O2}	0.43
O4→V_{O2}	1.89

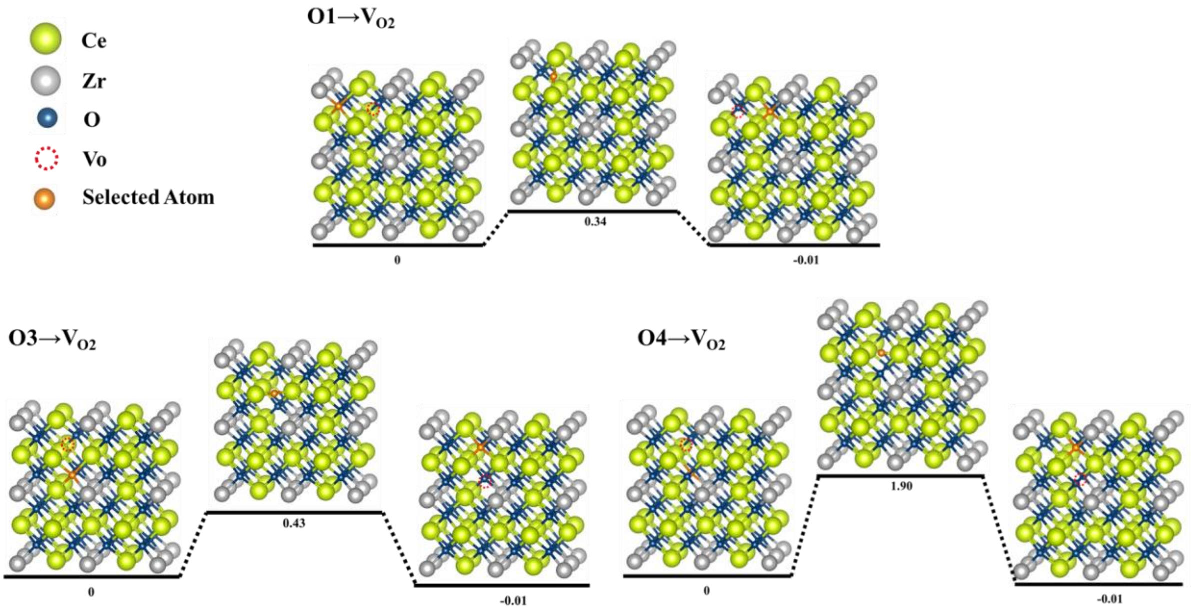

Figure S18. Energy potential profile along the O1→V_{O2}, O3→V_{O2} and O4→V_{O2} pathways in $Ce_{0.75}Zr_{0.25}O_{2-\delta}$ bulk. Among these, the O1→V_{O2} is the most favorable one.

S2.2 Model construction, oxygen formation energy and migration barrier calculations of $Ce_{0.75}Zr_{0.25}O_{2-\delta}$ (111) surface

When it comes to the reaction substrate, the research of Skorodumova et al. [3] suggested that the surface energy of the CeO_2(111) is low than that of CeO_2(110) and CeO_2(100) theoretically and experimentally, indicating that CeO_2 (111) surface is the most stable one. The atomic model of CeO_2 (111) and $Ce_{0.75}Zr_{0.25}O_2$ (111) surface can be directly cleaved from the CeO_2 and $Ce_{0.75}Zr_{0.25}O_2$ bulk model. Subsequently, the 4-layer 2×2 surface models of both CeO_2 (111) and $Ce_{0.75}Zr_{0.25}O_2$ (111) are used to avoid artificial interactions induced by the periodic boundary condition as shown in Figure S18. During the DFT calculation, the two bottom layers are restricted while the top two are relaxed. In this model, there are four non-equivalent surface oxygen atom sites, namely O_A, O_B, O_C and O_D. The computed $E_{O\_Formation}$ of all sites are lower than that of the pristine CeO_2 (111) (2.56 eV). Then we move on to study the migration of adjacent sub-surface oxygens to O_D vacancy sites, which hold the lowest oxygen vacancy formation energy of 2.08 eV (see in Table S5). The corresponding reaction energies of all possible O migration pathways are listed in Table S6, which the location of Oa, Ob, and Oc shown in Figure S18(b). The energy potential surfaces of the migration pathways are further studied as shown in Figure S19. The most favorable pathway (Oc → O_D) has a very low barrier of 0.376 eV, which is much lower than CeO_2 (111) surface (0.47 eV).

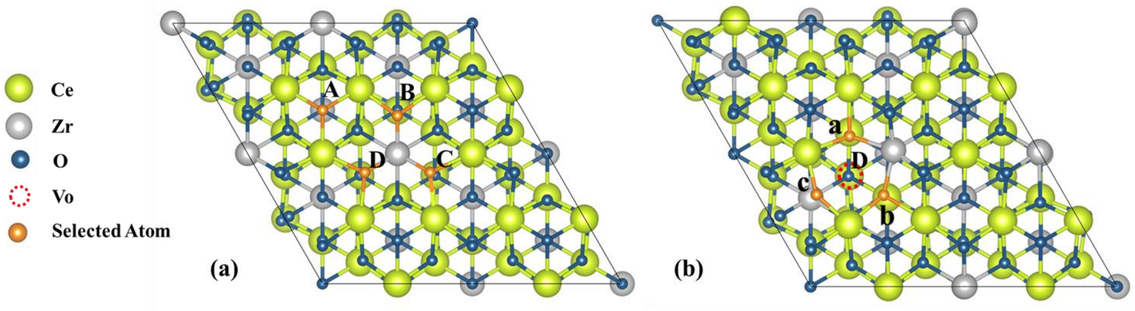

Figure S19. Model Structures of (a) $Ce_{0.75}Zr_{0.25}O_2$ (111) surface, (b) $Ce_{0.75}Zr_{0.25}O_{2-\delta}$ (111) surface with Vo at O_C site.

Table S5. Computed formation energy of each oxygen vacancy in $Ce_{0.75}Zr_{0.25}O_{2-\delta}$.

Vacancy Site	$E_{O\_Formation}$ (eV)
O_A	2.50
O_B	2.29
O_C	2.29
O_D	2.08

Table S6. Computed migration barrier of each oxygen migration to O_2 in $Ce_{0.75}Zr_{0.25}O_{2-\delta}$.

Migration pathway	E_m (eV)
Oa→V_{O_C}	0.41
Ob→V_{O_C}	0.39
Oc→V_{O_C}	0.23

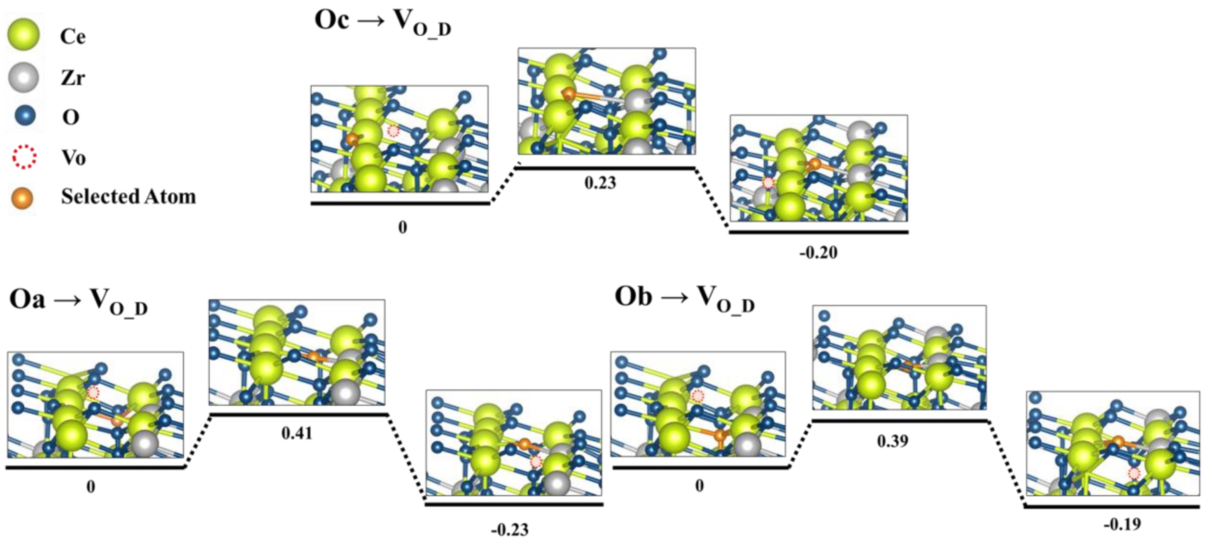

Figure S20. Energy potential profile along the Oa→V_{O_D}, Ob→V_{O_D} and Oc→V_{O_D} pathways on $Ce_{0.75}Zr_{0.25}O_{2-\delta}$ (111) surface. Among these, the Oc→V_{O_D} is the most favorable one.

S2.3 The adsorption of excited state CO_2* on the $Ce_{0.75}Zr_{0.25}O_{2-\delta}$ (111) surface

The adsorption of excited state CO_2^* on $Ce_{0.75}Zr_{0.25}O_{2-\delta}$ (111) surface is firstly searched on the base of the optimal ground state CO_2 adsorption, which turns out to be a linear geometry adsorption (Figure 6). The O vacancy has been proved to be the active site of the CO_2 dissolution reaction. Thus, the excited state CO_2^* is manually set to substitute the optimized ground state CO_2 adsorption site by replacing the C atoms and two O atoms respectively, with the geometry bent structure of excited state CO_2^* contained. The substrates are totally relaxed to reach the optimal structure. The reaction mechanism is well illustrated in Figure 6 with geometric properties listed in Table S7.

Table S7 Geometric Properties (explained in Figure 6) of ground state and excited state CO_2^* dissolution.

	$d_{C\text{-}O1}$	$d_{C\text{-}O2}$	$d_{O2\text{-}Zr}$	$d_{C\text{-}Zr}$	α_{OCO}
IS-V_O-e	1.26	1.26	2.83	-	118.27
IS-V_O-g	1.17	1.17	2.78	-	178.40
TS-V_O-g	1.36	1.89	-	2.71	-
FS-V_O-g	1.15	2.72	-	2.58	-

S2.4 The adsorption of O_2/O and CO_2 on the $Ce_{0.75}Zr_{0.25}O_{2-\delta}$ (111) surface

We've calculated the adsorption energy of O_2 and O to the active site (the oxygen vacancy) and found that both O_2 and O are more prone to adsorption than CO_2 (**Table S8**).

Table S8 Computed adsorption energy of intermediates on $Ce_{0.7}Zr_{0.3}O_2$ (111) surface

Intermediate	E_{ads} (eV)
O_2	-2.28
O	-2.65
CO_2	-0.94

REFERENCES

1. https://fr.lxcat.net/home/.

2. Pugachevskii, M. A.; Chibisov, A. N.; Mamontov, V. A.; Kuzmenko, A. P., Antioxidant Properties of Stabilized CeO_2 Nanoparticles. *physica status solidi (a)* **2021,** *218* (20).

3. Skorodumova, N. V.; Baudin, M.; Hermansson, K., Surface properties of CeO_2 from first principles. *Physical Review B* **2004,** *69* (7).
